# Transcriptional and physiological data reveal the dehydration memory behavior in switchgrass (*Panicum virgatum* L.)

**DOI:** 10.1186/s13068-018-1088-x

**Published:** 2018-04-02

**Authors:** Chao Zhang, Xi Peng, Xiaofeng Guo, Gaijuan Tang, Fengli Sun, Shudong Liu, Yajun Xi

**Affiliations:** 1 College of Agronomy, Northwest A&F University, Yangling, 712100 Shaanxi China; 2 State Key Laboratory of Crop Stress Biology for Arid Areas, Northwest A&F University, Yangling, 712100 Shaanxi China; 3 College of Plant Protection, Northwest A&F University, Yangling, 712100 Shaanxi China

**Keywords:** Switchgrass (*Panicum virgatum* L.), Biomass and biofuel, Dehydration stress, Transcriptional profiles, Abscisic acid, Jasmonic acid, Lignin biosynthesis

## Abstract

**Background:**

Switchgrass (*Panicum virgatum* L.) is a model biofuel plant because of its high biomass, cellulose-richness, easy degradation to ethanol, and the availability of extensive genomic information. However, a little is currently known about the molecular responses of switchgrass plants to dehydration stress, especially multiple dehydration stresses.

**Results:**

Studies on the transcriptional profiles of 35-day-old tissue culture plants revealed 741 dehydration memory genes. Gene Ontology and pathway analysis showed that these genes were enriched in phenylpropanoid biosynthesis, starch and sucrose metabolism, and plant hormone signal transduction. Further analysis of specific pathways combined with physiological data suggested that switchgrass improved its dehydration resistance by changing various aspects of its responses to secondary dehydration stress (D2), including the regulation of abscisic acid (ABA) and jasmonic acid (JA) biosynthesis and signal transduction, the biosynthesis of osmolytes (l-proline, stachyose and trehalose), energy metabolism (i.e., metabolic process relating to photosynthetic systems, glycolysis, and the TCA cycle), and lignin biosynthesis. The transcriptional data and chemical substance assays showed that ABA was significantly accumulated during both primary (D1) and secondary (D2) dehydration stresses, whereas JA accumulated during D1 but became significantly less abundant during D2. This suggests the existence of a complicated signaling network of plant hormones in response to repeated dehydration stresses. A homology analysis focusing on switchgrass, maize, and *Arabidopsis* revealed the conservation and species-specific distribution of dehydration memory genes.

**Conclusions:**

The molecular responses of switchgrass plants to successive dehydration stresses have been systematically characterized, revealing a previously unknown transcriptional memory behavior. These results provide new insights into the mechanisms of dehydration stress responses in plants. The genes and pathways identified in this study will be useful for the genetic improvement of switchgrass and other crops.

**Electronic supplementary material:**

The online version of this article (10.1186/s13068-018-1088-x) contains supplementary material, which is available to authorized users.

## Background

Switchgrass (*Panicum virgatum* L.) is a warm season, cross-pollinated, perennial C4 grass that is one of the most important lignocellulosic biofuel feedstock and pastures [1–3]. It belongs to the family Gramineae, and has two ecotypes: a lowland ecotype (tetraploid) and an upland ecotype (hexaploids and octoploids) [4]. Because of its rapid biomass production, cellulose-richness, easy degradation to ethanol, and the availability of extensive genomic information, switchgrass is a model bioenergy plant for research and is widely used for bioethanol production [5–9].

The perennial switchgrass has robust roots, exuberant tillers, and excellent resistance to drought, cold, heat, and biotic stresses [3, 10]. However, it is propagated by seed in large-scale plantations, and the seedlings are sensitive to water-deficit stress because of their slow development and weak roots, especially in arid, salinized or desertified regions. To improve the stress resistance of seedlings and create new varieties, it is important to understand the physiological responses and molecular mechanisms that are activated in switchgrass seedlings exposed to multiple drought stresses.

Previous studies on the drought stress responses of switchgrass seedlings have focused on morphological and physiological indices; a little is known about the response mechanisms at the molecular level. The water potential, gas exchange rates and photochemical traits of switchgrass leaves declined significantly during drought stress, and their drought tolerance correlated with an increase in the levels of reactive oxygen species induced by abscisic acid (ABA) [11, 12]. The drought tolerances of 49 switchgrass genotypes have been assessed using physiological and morphological parameters, revealing that drought-tolerant genotypes tend to have higher levels of ABA, spermine, trehalose, and fructose [13]. In addition, microRNAs have been shown to play important roles in switchgrass responses to drought stress, which involved the biosynthesis of carbon compounds, glucose, starch, fatty acid and lignin [14]. In particular, miR156 miR162 exhibited pronounced changes in expression under severe drought stress, suggesting that they may contribute to the acclimatization process [15].

The dissection of dehydration stress response mechanisms at the molecular level is required to support genetic improvement and germplasm creation by subtly regulating pathways involved in the responses of interest. Numerous studies have shown that ABA plays key roles in plant drought stress responses. Two classes of drought-related signaling pathways have been identified: ABA-dependent and ABA-independent [16, 17]. ABA is synthesized from mevalonic acid in plants; the key enzyme in the process is 9-*cis*-epoxycarotenoid dioxygenase (NCED) [18]. ABA is degraded by abscisic acid 8′-hydroxylase (CYP707A3) [19] and ABA glucosyltransferase (ABA GTase) [20]. Proteins involved in ABA-dependent signaling pathways include protein phosphatase 2C (PP2C), ABA-responsive element binding factor (AREB/ABF), NAC transcriptional factor (RD26), and the downstream elements *RD22* (responsive to dehydration 22), *RD29B*, *RD20A* [17, 21–23]. ABA-independent signaling proteins include members of the HD-ZIP and DREB2 families, as well as the downstream components early responsive to dehydration 1 (*ERD1*) and *RD29A*, among others [17, 24–26]. Most modern efforts to identify genetic modifications that improve plant drought resistance involve these two pathways.

However, there is another set of genes that respond to multiple dehydration stress but which have been neglected for a long time. These genes were named dehydration stress memory genes (DMGs) because they exhibit a “memory” of previous dehydration stresses [27, 28]. The expression of DMGs can be strongly and rapidly induced or suppressed when plants experience secondary dehydration [28]. Stress memory has been observed in many species, including *Arabidopsis thaliana* [28–30], *Zea mays* [31], *Brassica rapa* [32], *Solanum tuberosum* [33] and *Aptenia cordifolia* [34, 35]. These transcriptional memory genes are closely intertwined with histone methylation, plant hormone signaling, and osmotic adjustment [28, 34, 36–40]. In *Arabidopsis* and maize, four types of DMGs were identified using high-throughput RNA sequencing (RNA-Seq) [30, 31]. DMG expression allows plants to strongly and rapidly respond to repeated or long-term intermittent dehydration stresses and improves their survival rate and biomass [41].

To identify DMGs, explore their formation networks, and establish a firmer foundation for genetic improvement, we analyzed the response patterns of switchgrass plants subjected to repeated dehydration stresses. Switchgrass is a cross-pollinated plant, so tissue-cultured plants were used in this work to eliminate the effects of seedling genotypes. Transcriptomic data were acquired using RNA-Seq, and DMGs and regulatory networks were identified and analyzed carefully based on annotations and published papers. Physiological indices, chemical substance assays and transcriptional information suggested that switchgrass improved its response to secondary dehydration stress by changing the pathways of ABA and jasmonic acid (JA) biosynthesis and signal transduction, increasing the biosynthesis of osmolytes (l-proline, raffinose, stachyose and trehalose), and modifying its energy metabolism systems (specifically, those involved in photosynthesis, glycolysis, and the tricarboxylic acid cycle). Lignin biosynthesis, the persistence of the transcriptional memory, and the conservation and species-specificity of DMGs in switchgrass, maize and *Arabidopsis* were also analyzed.

## Methods

### Plant material and experimental design

**Fig. 1:**
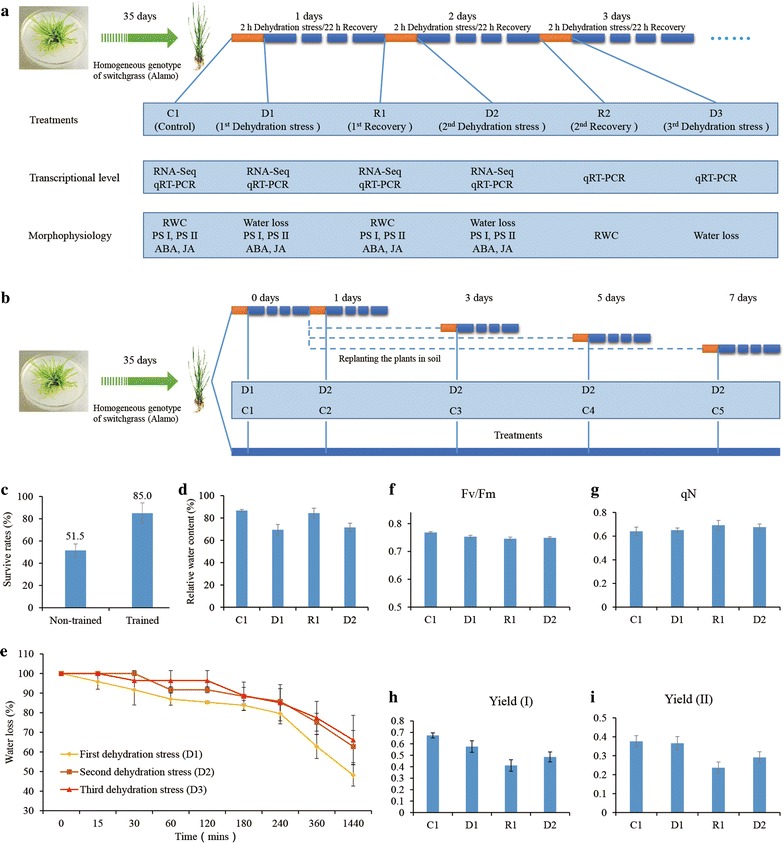
Experimental design and physiological assays. **a** Experimental design for repeated dehydration stresses and sample collection. **b** Experimental design for determining the persistence of dehydration memory and sample collection. **c** Survival rates of trained and non-trained switchgrass. **d** Relative water content of leaves during repeated dehydration stresses. **e** Water loss from leaves during the first, second and third dehydration stresses. **f**–**i**, Determination of photosynthetic activity during repeated dehydration stresses. *C* control, *D1*–*3* the first, second, and third dehydration stresses, *R1*–*2* the first and second recovery periods, *RNA-Seq* RNA sequencing, *qRT-PCR* quantitative real-time PCR, *RWC* relative water content, *PS I and PS II* photosynthesis system I and II, *ABA* abscisic acid, *JA* jasmonic acid, *Fv/Fm* maximum photosynthetic potential, *qN* non-photochemical quenching, *Yield* (*I*) *and Yield* (*II*) real-time quantum yield of PSI and PSII. Values for RWC, water loss and photosynthesis parameters are means of *n* = 5 measurements ± SD

Switchgrass is allogamous and has strong genetic self-incompatibility, so it is difficult to create homogenous materials. In this study, we acquired a homogeneous genotype of switchgrass by in vitro tissue culture from axillary buds of a single plant belonging to the lowland Alamo cultivar (introduced from the USA and domesticated at Northwest A&F University, China). The regenerated shoots were proliferated in MS medium supplemented with 13.3 μM L^−1^ 6-benzylaminopurine (BAP) [42]. Cluster shoots were separated and transplanted to substrates of sand and soil (1:1) in a greenhouse illuminated with natural sunlight (day/night ~ 14/10 h; daily temperature 28–32 °C). Multiple dehydration stresses were imposed based on designs previously used with *Arabidopsis* [28, 30] and maize [31] (Fig. 1a). Five-week-old homogenous plants with three fully expanded leaves were removed from their substrates, washed to remove residual soil, and then acclimated overnight in a plant growth chamber with their roots in water. The next morning, the plants were blotted onto filter paper to remove water, and air-dried for 2 h (this corresponded to the first dehydration stress, D1). At the same time, non-stressed control plants designated C1 were sampled as controls. The drying was followed by a 22 h recovery period of full re-hydration at 25 °C (first recovery, R1). For the subsequent stress treatment, R1 plants were air-dried for 2 h (the second dehydration stress, D2) followed by another recovery period (second recovery, R2). The same procedures were repeated for D3, R3, and so on (Fig. 1a). To determine the duration of the transcriptional memory, stressed plants were replanted in soil for 3, 5, or 7 days before being re-exposed to dehydration stress [28] (Fig. 1b).

### RNA isolation and RNA-Seq library construction

Second fully expanded leaves were collected for RNA isolation and for physiological and biochemical assays. The collected leaves were immediately frozen in liquid nitrogen and stored at − 80 °C. Total RNA was extracted with the Trizol reagent (Invitrogen Life Technologies, USA) according to a published protocol [43]. The isolated RNA was subsequently treated with RNase-Free DNase I (Takara, http://www.takarabiomed.com.cn/). The quantity, purity and integrity of the resulting RNA was confirmed using NanoDrop One (Thermo Scientific, USA) and Agilent 2100 (Agilent Technologies, USA) instruments; only RNAs classified as A-type (optimal RNA samples for RNA sequencing, BGI-Shenzhen, http://www.genomics.cn/) were used for cDNA library construction.

Two biological replicates of four treatments were sequenced (C1, D1, R1 and D2, Fig. 1a), giving a total of eight RNA sequencing (RNA-Seq) samples. Each treatment contained more than 12 plants. Total RNA was performed according to the standard transcriptome resequencing experimental process, and was sequenced using the Illumina GAII platform (HiSeq2500) at BGI-Shenzhen [43]. Expressed tags were calculated and normalized to FPKM (Fragments Per Kilobase of exon per Million fragments mapped) values for gene expression analysis using the Cufflinks program [44]. An overview of the RNA-Seq process and data on the repeatability of different biological replicates are provided in Additional file 1: Figure S1. The raw sequencing files of transcriptomic data were uploaded to sequence read archive (SRA) at NCBI (Accession Number PRJNA394165).

### Bioinformatics analysis

Genes in the transcriptomic profiles were predicted using data from the switchgrass genomic database (https://phytozome.jgi.doe.gov/pz/portal.html#!info?alias=Org_Pvirgatum) [8]. Differentially expressed genes (DEGs) were identified using the Cuffdiff [44] tool from the Cufflinks package (http://cole-trapnell-lab.github.io/cufflinks/) based on their FPKM values and the associated *q* values (false discovery rate adjusted *p* values) between different treatments. Specifically, a gene was identified as a DEG if it had a *q* value ≤ 0.05 and a | log (base2) fold change | ≥ 1. In addition, the gene’s FPKM value under the dehydration stress (for up-regulated genes) or control (for down-regulated genes) treatments was required to be at least 20% of the mean FPKM value for all expressed genes [45]. Genes exhibiting common or differential expression in different treatments were compared and identified using Venny 2.1 (http://bioinfogp.cnb.csic.es/tools/venny/index.html).

Dehydration stress memory genes (DMGs) were identified by comparing expressed fold changes between D1/C1 and D2/D1 within the DEG libraries. Genes that were up-regulated, down-regulated, and exhibited no significant change in expression are indicated by the symbols “+”, “−” and “=”, respectively. Changes in an individual gene’s expression are denoted in the format [*a*/*b*], where *a* is the symbol indicating the change between C1 and D1, and *b* is the symbol indicating the change between D1 and D2. Thus, for example, a gene of the [+/+] type was up-regulated between C1 and D1, and also between D1 and D2, whereas a gene of the [=/+] type exhibited no change in expression between C1 and D1, but was up-regulated between D1 and D2 [30, 31]. Eight types of DEGs were defined [+/+], [+/−], [−/+], [−/−], [+/=], [−/=], [=/+] and [=/−] (Table 1). The first four types were considered to be DMGs because their responses changed between D1 and D2. The [+/=] and [−/=] genes were considered non-memory genes, and [=/+] and [=/−] genes were defined as late-response genes. A list of all identified DMGs is provided in Additional file 2: Table S1.

**Table 1:** Dehydration response and transcriptional memory genes in switchgrass, maize and *Arabidopsis*

	Switchgrass	Maize	*Arabidopsis*
Total genes by RNA-Seq	47,207	39,635	33,555
Dehydration response	1566 (3.3%)	2062 (5.2%)	6579 (19.6%)
Induced	811	1636	3396
Repressed	755	426	3183
Non-memory genes	825	1246	4616
Induced [+/=] (C1 < D1 = D2)	337	941	2177
Repressed [−/=] (C1 > D1 = D2)	488	305	2439
Memory genes	741	816	1963
[+/+] (C1 < D1 < D2)	93 (13%)	162 (20%)	362 (18%)
[−/−] (C1 > D1 > D2)	113 (15%)	72 (9%)	310 (16%)
[+/−] (C1 < D1 > D2)	381 (51%)	533 (65%)	857 (44%)
[−/+] (C1 > D1 < D2)	154 (21%)	49 (6%)	434 (22%)
Late-response genes	5639 (11.9%)	2924 (7.4%)	1371 (4.1%)
[=/+] (C1 = D1 < D2)	3158	1678	798
[=/−] (C1 = D1 > D2)	2860	1246	573

Genes that were up-regulated, down-regulated, and exhibited no significant change in expression are indicated by the symbols “+”, “−” and “=”, respectively. Changes in an individual gene’s expression are denoted in the format [*a*/*b*], where *a* is the symbol indicating the change between C1 and D1, and *b* is the symbol indicating the change between D1 and D2. Thus, for example, a gene of the [+/+] type was up-regulated between C1 and D1, and also between D1 and D2, whereas a gene of the [=/+] type exhibited no change in expression between C1 and D1, but was up-regulated between D1 and D2

All the identified switchgrass genes were annotated using the NCBI Blast tools (version 2.2.8) in conjunction with the NCBI non-redundant protein sequences (nr) database, the *Arabidopsis* Information Resource Proteins database (release TAIR10), the maize B73 RefGen_v3 database, and the Rice Genome Annotation Project (RGAP) release 7. Gene Ontology (GO) analysis was performed using AgBase GORetriever and GOSlimViewer [46], and pathways were enriched using the NCBI Flink (https://www.ncbi.nlm.nih.gov/Structure/flink/flink.cgi) and KEGG (Kyoto Encyclopedia of Genes and Genomes) databases. Specific pathways were verified using Blastp in conjunction with the switchgrass genomic database and the TAIR10 database. Orthologous genes belonging to larger families were analyzed with reference to previously published works.

### Reverse transcription and quantitative real-time PCR

Total RNA was reverse-transcribed into cDNA using the PrimeScript™ RT reagent Kit with the gDNA Eraser (RR047A, Takara) according to the standard protocol. cDNA was detected using two housekeeping genes, *PvACT2* (PvActin 2) [47] and *PveEF*-*1α* (Eukaryotic elongation factor 1α) [48]. Quantitative real-time PCR (qRT-PCR) was performed with QuantStudio™ 7 Flex Real-Time PCR System (Thermo Scientific, USA) using SYBR Premix Ex Taq™ II Kit (RR820A, Takara) with three technical replicates. Relative gene expressed levels were calculated using the ΔΔ*C*_T_ method with *PveEF*-*1α* as the internal control, and three biological replicates were performed for each experiment. The primers used in the qRT-PCR experiments were designed using Primer Premier 6 (http://www.premierbiosoft.com/primerdesign/index.html), and are listed in Additional file 3: Table S2.

### Physiological indices and chemical substance assays

The plants used to determine the survival rates of stress trained or non-stress-trained plants were grown in substrates of sand and soil (1:1) in greenhouses as described in the plant material section. Relative water content (RWC) was calculated using the equation ${\text{RWC }}\left( \% \right) = [({\text{fresh}}\,{\text{weight}} - {\text{dry}}\,{\text{weight}})/({\text{turgid}}\,{\text{weight}}\, -\, {\text{dry}}\,{\text{weight}})] \times 100\%$ [28]. The water loss was determined by measuring the leaves’ weight at a fixed time interval after their detachment from the plants [28]. The Photosynthesis System I and II (PSI and PSII) parameters were measured with a DUAL-PAM-100 fluorometer (WALZ, Germany) by synchronously recording changes in P700 (PSI) and chlorophyll fluorescence (PSII) [49]. The contents of abscisic acid (ABA) and jasmonic acid (JA) in the leaves of plants subjected to repeated dehydration stress were determined by high performance liquid chromatography–mass spectrometry (HPLC–MS) with an Agilent 1290 Infinity II liquid chromatograph (Agilent Technologies, USA) and a QTRAP 6500 MS/MS System (AB SCIEX, USA). Reference standards (ABA and JA, LC grade) were bought from Sigma-Aldrich (USA). Proline was extracted from leaves with 3% sulfosalicylic acid, and then reacted with acid ninhydrin reagent. After extracting with methylbenzene, absorbances at 520 nm were measured to calculate proline content.

Five biological replicates were used in the determination of RWC, water loss and photosynthesis parameters, and three biological replicates were used for hormone determination. Each biological replicate involved at least 12 plants, and only the second fully expanded leaves were collected for the experiments. All data were analyzed with the Duncan test [50] using the SAS 9.2 software (http://www.sas.com/en_us/software/sas9.html).

## Results

### Physiological indices indicate that switchgrass exhibits dehydration memory

To determine whether switchgrass plants exhibit stress memory after a previous dehydration stress, we designed multiple dehydration stress experiments and measured the plants’ survival rates, leaf relative water content (RWC) and water loss as the experiments progressed (Fig. 1a). The survival rates of trained plants were significantly higher than those for non-trained plants (Fig. 1c), and the plants that had experienced dehydration stress also exhibited slower water loss than non-trained plants (Fig. 1e). The RWC of the leaves after the first recovery (R1) and second recovery (R2) periods was similar to that in control plants (Fig. 1d), indicating that the plants recovered fully from the previous dehydration stress during the recovery periods, and started each new stress period with a similar RWC. In addition, there were significant changes in the plants’ survival rates and water losses during successive dehydration periods, indicating that the training had improved the plants’ dehydration stress resistance and that the trained plants exhibited stress memory.

To validate this conclusion, selected photosynthetic parameters were measured for PSI and PSII, namely Fv/Fm, non-photochemical quenching (qN), Yield (I) and Yield (II). These parameters reflect the maximum photosynthetic potential of the leaves, the photoprotective capacity of the leaves, and the real-time quantum yields of PSI and PSII, respectively [51]. There were no appreciable differences in Fv/Fm or qN between plants during the first dehydration stress (D1) and the corresponding controls (C1), or between plants during the first recovery period (R1) and the second dehydration stress (D2) (Fig. 1f, g). However, Yield (I) and Yield (II) during R1 and D2 were significantly lower than in C1 and D1. These results indicated that switchgrass underwent appreciable changes after the first dehydration stress even though the mild stresses imposed in this work were not lethal.

### Identification of dehydration memory genes (DMGs) in switchgrass

To characterize the mechanisms responsible for dehydration memory in switchgrass, the transcriptomes of C1, D1, R1, and D2 plants were studied by high-throughput RNA sequencing (Fig. 1a). 98,007 genes have been identified according to switchgrass genome, and 47,207 genes (48.17%) were expressed in the transcriptomes. The average proportion of clean reads in all samples was 97.14%, and the repetitive rates of gene expression numbers across two biological replicates were 89.73% (C1), 87.77% (D1), 92.40% (R1) and 91.08% (D2) (Additional file 1: Figure S1). Differentially expressed genes (DEGs) were identified as genes for which *q* ≤ 0.05, | log (base2) fold change | ≥ 1, and the FPKM value under dehydration stress (for up-regulated genes) or in the control treatment (for down-regulated genes) was at least 20% of the mean FPKM value for all expressed genes [45]. The coefficient values of DEGs in two biological replicates were 98.66% (C1), 99.23% (D1), 99.78% (R1) and 98.67% (D2) (Additional file 1: Figure S1), demonstrating the excellent repeatability of the results across biological replicates.

Analysis of the expressed fold changes for DEGs between D1 and C1, and between D2 and D1, revealed four types of DMGs, two types of non-memory genes, and two types of late-response genes in switchgrass. Switchgrass has more detected genes than *Arabidopsis* or maize, but its number of dehydration response genes (1566) is lower than that determined for either species (2062 in maize, and 6759 in *Arabidopsis*) (Table 1). The number of transcriptional memory genes in switchgrass (741) was similar to that in maize (816), but significantly lower than that in *Arabidopsis* (1963). The non-memory genes exhibited similar trends in all three species. Interestingly, switchgrass has twice as many late-response genes as maize, and three times as many as *Arabidopsis* (Table 1).

**Fig. 2:**
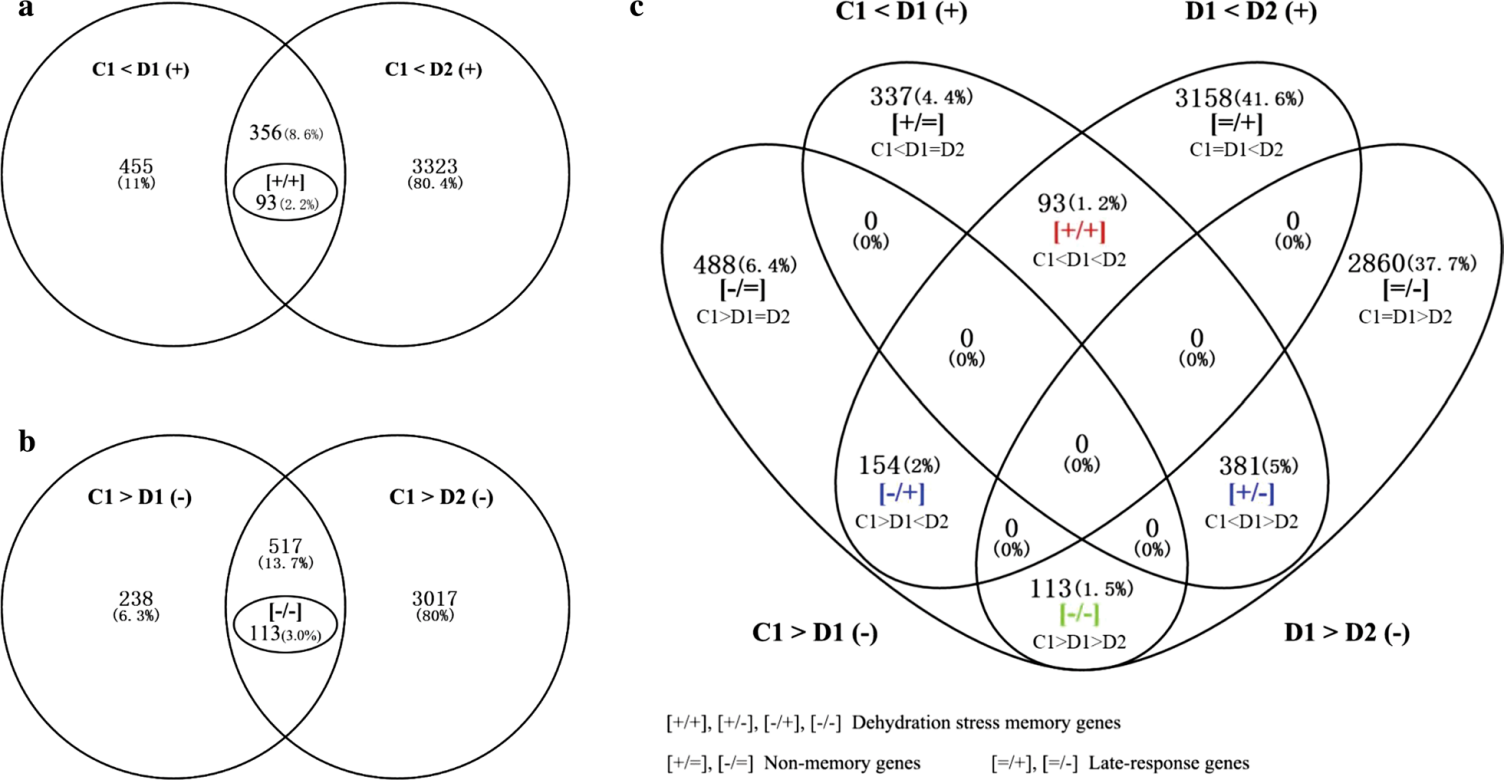
**a** Venn diagrams of up-regulated genes in D1 and D2. **b** Venn diagrams of down-regulated genes in D1 and D2. **c** Venn diagrams of different types of dehydration memory genes. *C* control, *D1*–*2* first and second dehydration stresses, *R1* first recovery period, “+”, “−” and “=” denote up-regulated genes, down-regulated genes, and genes with no significant changes in expression, respectively

The memory types of switchgrass dehydration stress response genes were revealed by constructing Venn diagrams (Fig. 2). There were 811 and 3679 genes up-regulated in D1 and D2, respectively, of which 356 were common genes (i.e., genes up-regulated in both dehydration periods). Ninety-three of the memory genes were [+/+] memory genes, corresponding to 26.12% of the common up-regulated genes and 2.2% of all up-regulated genes (Fig. 2a). In addition, there were 755 and 3534 genes that were down-regulated in D1 and D2, respectively, and 517 common genes that were down-regulated in both dehydration periods. One hundred and thirteen of these genes were [−/−] memory genes, corresponding to 21.86% of the common down-regulated genes and 3.0% of all down-regulated genes (Fig. 2b). Moreover, 381 [+/−] memory genes and 154 [−/+] memory genes were identified, corresponding to 5 and 2% of all DEGs (7205), respectively (Fig. 2c). Finally, 337 (4.4%) [+/=] type and 488 (6.4%) [−/=] type non-memory genes were identified, along with 3158 (41.6%) [=/+] type and 2860 (37.7%) [=/−] type late-response genes (Fig. 2c). The numbers of [=/+] and [=/−] late-response genes identified in switchgrass were both significantly higher than the corresponding numbers for maize and *Arabidopsis* (Table 1). Among the dehydration memory genes, [+/−] memory genes were significantly more abundant than the other three types, accounting for 51% of all DMGs in switchgrass, 65% in maize, and 44% in *Arabidopsis* (Table 1).

### Verification of DMGs by quantitative real-time PCR (qRT-PCR)

**Fig. 3:**
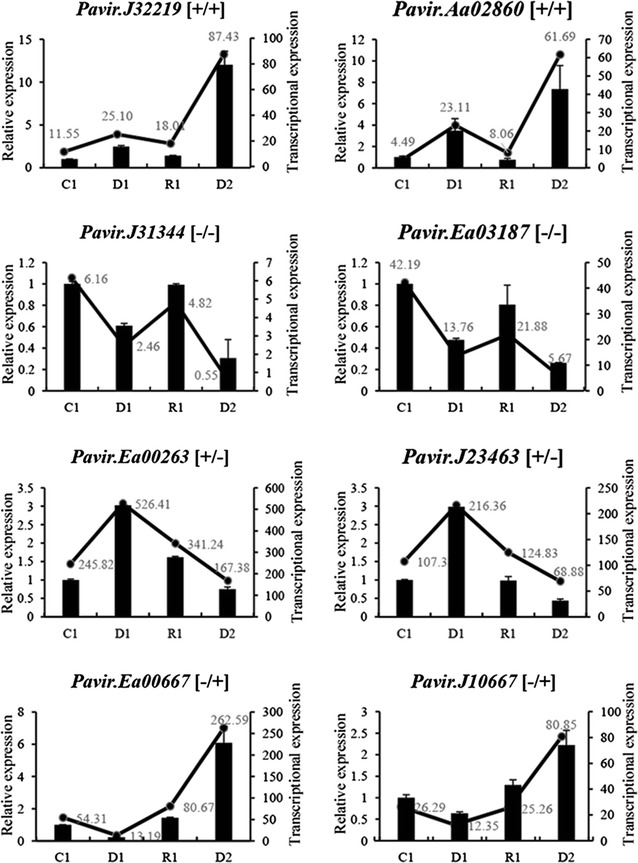
Verification of four types of dehydration memory genes by quantitative real-time PCR. *C* control, *D1*–*2* first and second dehydration stresses, *R1* first recovery period. Relative gene expressed levels were calculated using the ΔΔ*C*_T_ method with *PveEF*-*1α* as the internal control, and three biological replicates were performed for each experiment

The RNA-Seq results were validated using qRT-PCR to quantify the expression of two randomly selected genes from each DMG types, making a total of 8 genes or 32 treatments: *Pavir.J32219* and *Pavir.Aa02860* representing the [+/+] type, *Pavir.J31344* and *Pavir.Ea03187* representing the [−/−] type, *Pavir.Ea00263* and *Pavir.J23463* representing the [+/−] type, and *Pavir.Ea00667* and *Pavir.J10667* representing the [−/+] type. The measured expression of all eight genes was entirely consistent with the transcriptomic data (Fig. 3), indicating that the transcriptomic approach based on RNA-Seq is a reliable method for analyzing the response of switchgrass to multiple dehydration stresses.

### Functional analysis of switchgrass DMGs

**Fig. 4:**
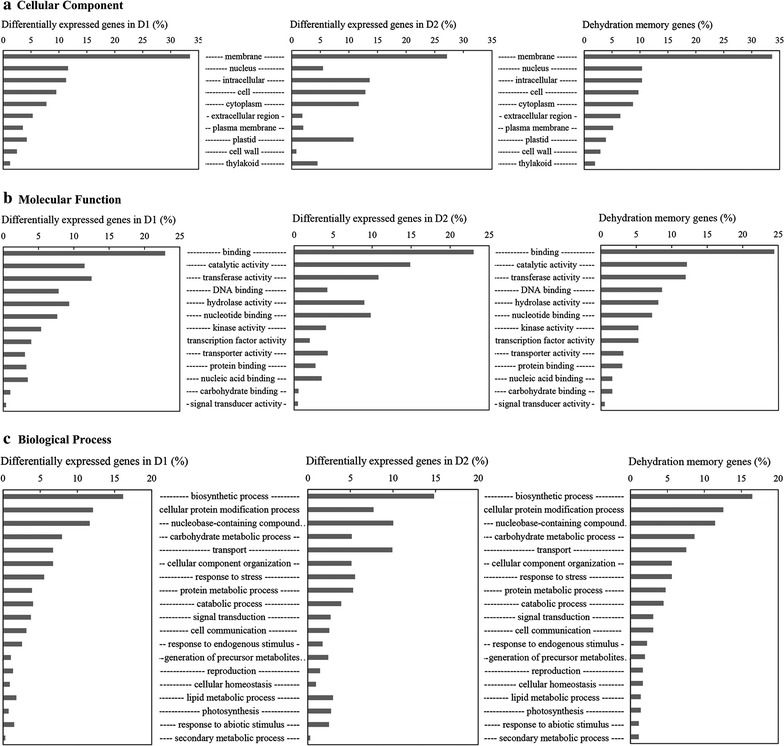
Gene Ontology analysis of dehydration memory genes with differentially expressed genes in D1 and D2. **a** Cellular component. **b** Molecular function. **c** Biological process. D1–2, first and second dehydration stresses

To explore the underlying mechanism of dehydration memory, all the DMGs were annotated using Blastp in conjunction with the NCBI (nr) protein database and species-specific databases for *Arabidopsis* (TAIR10), maize (B73 RefGen_v3), and rice (RGAP release 7) (Additional file 2: Table S1). The Gene Ontology (GO) annotations of DEGs in D1 and D2, and DMGs, were collected and used to construct graphs (Fig. 4). The chosen genes were enriched in the following Cellular Component GO terms: membrane (GO: 0016020), intracellular (GO: 0005634) and nucleus (GO: 0005622). They were also enriched in the binding (GO: 0005488), catalytic activity (GO: 0003824) and transferase activity (GO: 0016740) terms from the molecular function category (Fig. 4). The biological process annotations revealed enrichment in the terms biosynthetic process (GO: 0009058), cellular protein modification process (GO: 0006464) and nucleobase-containing compound metabolic process (GO: 0006139) in D1, while GO: 0009058, GO: 0006139 and transport (GO: 0006810) were enriched in D2. In addition to these four terms, the carbohydrate metabolic process (GO: 0005975), response to stress (GO: 0006950) and signal transduction (GO: 0007165) terms were also enriched among the DMGs (Fig. 4). Finally, the pathway enrichment analysis showed that phenylpropanoid biosynthesis (ko00940), starch and sucrose metabolism (ko00500), plant hormone signal transduction (ko04075), photosynthesis—antenna proteins (ko00196), galactose metabolism (ko00052) were enriched among the DMGs (Additional file 4: Table S3). These results indicated that dehydration stress memory in switchgrass involves plant hormone biosynthesis and signal transduction, osmotic adjustment (ko00500, ko00052, etc.), photosynthesis, and lignin biosynthesis (ko00940). Therefore, differentially expressed genes associated with these pathways were investigated.

### Plant hormone biosynthesis and signal transduction

#### Abscisic acid (ABA) biosynthesis and signal transduction

**Fig. 5:**
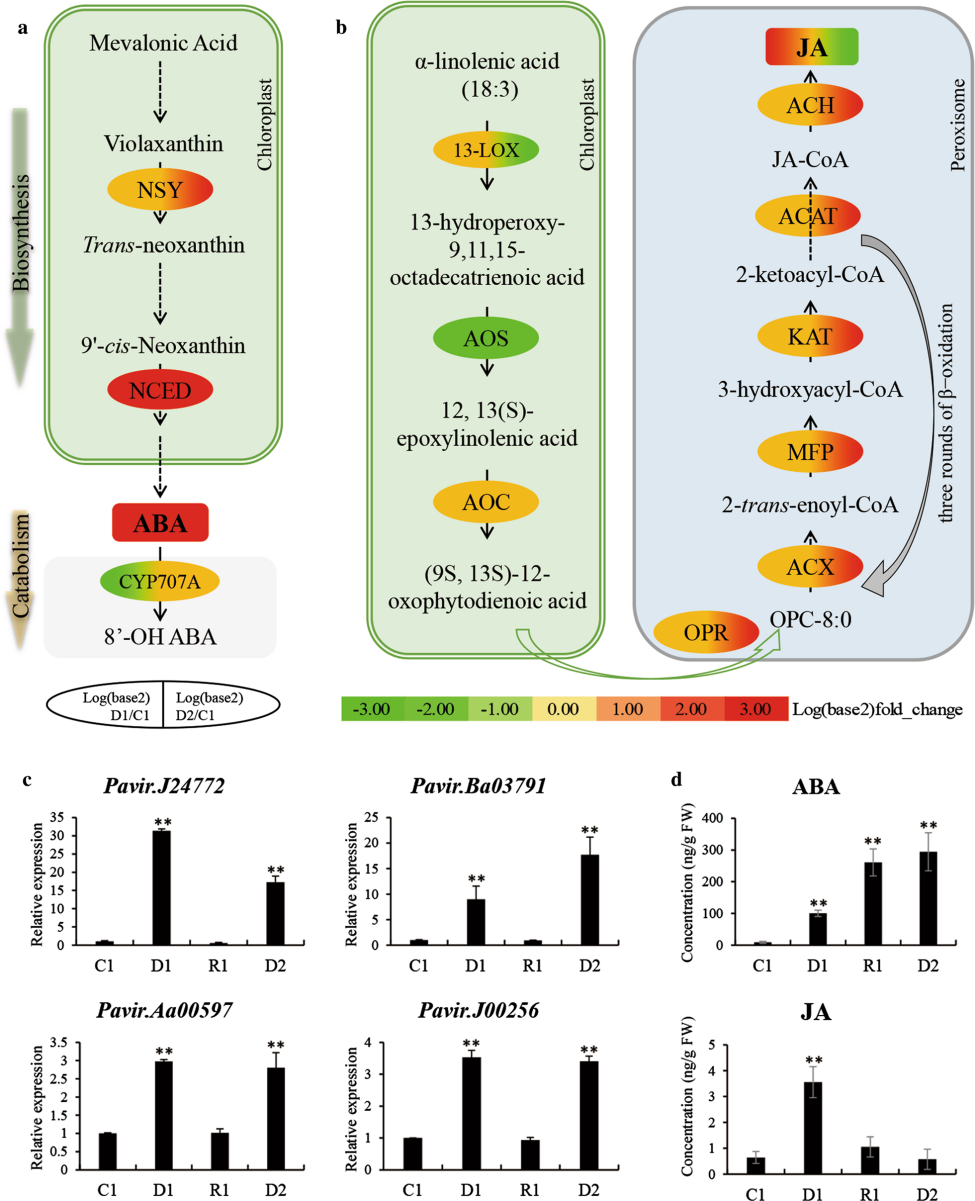
ABA and JA metabolism in multiple dehydration stresses. **a** ABA biosynthesis and catabolism. **b** JA biosynthesis. **c** Relative expression of orthologs of NCED and ABF. **d** ABA and JA concentrations in leaves determined by HPLC-MS. *ABA* abscisic acid, *JA* jasmonic acid, *NSY* neoxanthin synthase [EC 5.3.99.9], *NCED* 9-*cis*-epoxycarotenoid dioxygenase [EC 1.13.11.51], *CYP707A* [+]-abscisic acid 8′-hydroxylase [EC 1.14.13.93], *ABF* ABA responsive element binding factor, *13-LOX* linoleate 13S-lipoxygenase [EC 1.13.11.12], *AOS* allene oxide synthase [EC 4.2.1.92], *AOC* allene oxide cyclase [EC 5.3.99.6], *OPR* 12-oxophytodienoate reductase [EC 1.3.1.42], *ACX* acyl-CoA oxidase [EC 1.3.3.6], *MFP* multifunctional—protein enoyl-CoA hydratase [EC 4.2.1.17], *KAT* 3-ketoacyl-CoA thiolase [EC 1.1.1.35], *ACAT* acetyl-CoA C-acyltransferase [EC 2.3.1.16], *ACH* acyl-CoA hydrolase [EC 3.1.2.20], *FW* fresh weight. Relative expression values and plant hormone concentrations are means of *n* = 3 measurements ± SD (Duncan’s test: **p < 0.01)

Plant hormone biosynthesis and signal transduction (GO: 0007165; ko04075) were enriched in both the GO and pathway analyses (Fig. 4; Additional file 4: Table S3), and more than 80 DEGs were identified as being involved in ABA and jasmonic acid (JA) biosynthesis and signal transduction (Additional file 5: Table S4). ABA plays important roles in the drought stress response, contributing to signal perception and transduction, stomatal closure, and osmotic adjustment [20, 38, 52]. In switchgrass subjected to multiple dehydration stresses, four orthologs of AtNCEDs, the rate-limiting enzyme of ABA biosynthesis, were significantly up-regulated in D1 and D2. One of them (Pavir.Ba03791) was a [+/+]-type memory gene. In addition, two orthologs of CYP707A3 (Pavir.Ab02775 and Pavir.J08122), a key enzyme of ABA catabolism, were down-regulated in D1 and slightly down-regulated in D2 (Fig. 5a; Additional file 5: Table S4). The qRT-PCR relative expression analysis also showed that the orthologs of NCED (Pavir.J24772 and Pavir.Ba03791) were strongly up-regulated in D1 and D2 (Fig. 5c). The transcriptional data suggest that ABA accumulated during the dehydration stresses. Therefore, we determined the ABA contents of leaves in different treatments using HPLC–MS, confirming that this hormone accumulated significantly in D1, R1 and D2, with very large accumulations occurring in the latter two periods (Fig. 5d). These results strongly suggest that ABA plays a key role in the response of switchgrass to repeated dehydration stresses. Moreover, the qRT-PCR and HPLC–MS results verified the reliability of the initial transcriptional data.

**Fig. 6:**
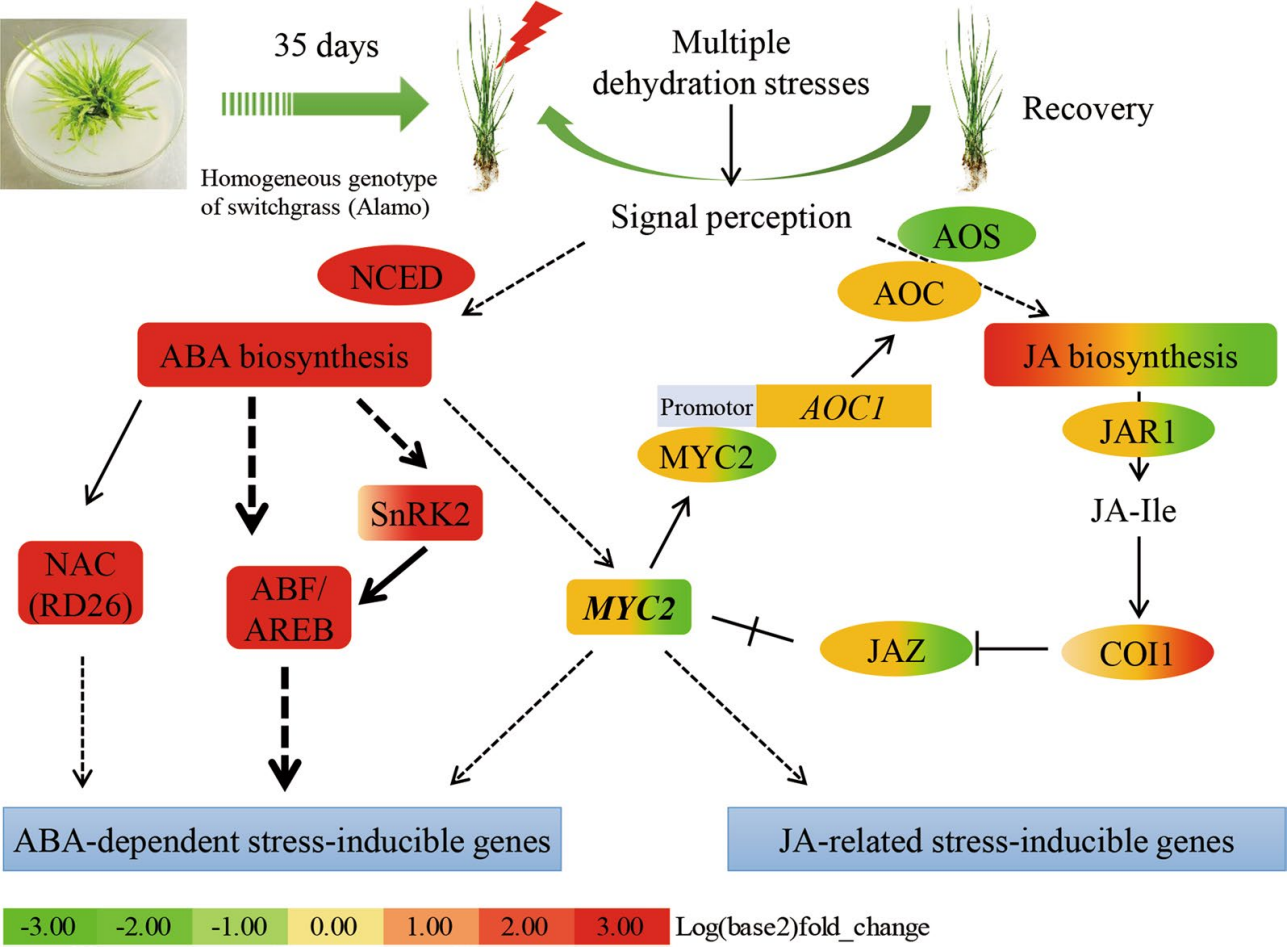
ABA and JA signal transduction in multiple dehydration stresses. *ABA* abscisic acid, *JA* jasmonic acid, *NCED* 9-*cis*-epoxycarotenoid dioxygenase [EC 1.13.11.51], *NAC* transcription factor NAC, *RD* responsive to desiccation, *ABF/AREB* ABA responsive element binding factor, *SnRK2* serine/threonine-protein kinase SRK2 [EC:2.7.11.1], *AOS* allene oxide synthase [EC 4.2.1.92], *AOC* allene oxide cyclase [EC 5.3.99.6], *JAR1* jasmonic acid-amino synthetase, *COI1* coronatine-insensitive protein 1, *JAZ* jasmonate ZIM domain-containing protein, *MYC2* transcription factor MYC2

Clade A protein phosphatase 2C (PP2CA) enzymes function as co-receptors in ABA signaling and respond positively to increases in ABA levels or stress-induced ABA biosynthesis [53, 54]. Among the downstream enzymes of the PP2Cs are subfamily 2 of the SNF1-related kinases (SnRK2s), which are positive regulators of ABA signal transduction [55, 56]. Our results identified nine PP2CA orthologs that were strongly up-regulated in D1 and D2, and four orthologs of SnRK2s that were up-regulated in D1 and D2, especially in D2 (Fig. 6; Additional file 5: Table S4). Among these genes, three PP2CA orthologs and one SnRK2 ortholog were [+/+] type memory genes. In addition, two orthologs (Pavir.Aa00597, Pavir.J00256) of ABA responsive element binding factors (ABFs) belonging to the ABA-dependent pathway were significantly up-regulated in D1 and D2 (Fig. 6; Additional file 5: Table S4). Both the transcriptional and qRT-PCR data indicated that these two genes functioned as non-memory genes ([+/=]) (Fig. 5c). In addition, two orthologs (Pavir.Ea02724, Pavir.Eb03036) of responsive to desiccation 22 (*RD22*) and one ortholog (Pavir.J02116) of ras-related protein Rab-18 were significantly up-regulated in D2 (Additional file 5: Table S4).

#### JA biosynthesis and signal transduction

JA is synthesized in the chloroplasts and peroxisomes, and a previous study demonstrated that it engages in crosstalk with ABA biosynthesis and signal transduction, and is also involved in biotic or abiotic stress responses [40, 57–61]. More than 50 of the DEGs identified in this work were annotated with JA biosynthesis and signal transduction terms (Additional file 6: Table S5). Twelve orthologs of upstream genes that function in the chloroplast (*LOX*, *AOS* and *AOC*) were down-regulated in D2, and 18 orthologs of downstream genes that function in the peroxisome (*OPR*, *ACX*, *MFP*, *KAT*, *ACAT* and *ACH*) were up-regulated in D2 (Fig. 5b; Additional file 6: Table S5). The HPLC–MS assay showed that JA accumulated significantly in D1 but returned to its initial level in R1 and D2 (Fig. 5d).

*JAR1*, *COI1*, and the JAZ transcription factors play important roles in JA signaling [58, 61]. In the present study, six orthologs of *JAR1* and five orthologs of the JAZ transcription factor were down-regulated during the multiple dehydration stresses, especially in D2, while the *COI1* orthologs Pavir.Ea03420 and Pavir.J07109 were strongly up-regulated in D2 (Fig. 6; Additional file 6: Table S5). Furthermore, two orthologs of MYC2, a basic helix-loop-helix (bHLH) transcription factor involved in *AOC* expression and the crosstalk between ABA and JA biosynthesis and signal transduction [40], were slightly up-regulated in D1 and down-regulated in D2. Another four orthologs of MYC2 were down-regulated in both D1 and D2 (Fig. 6; Additional file 6: Table S5).

#### Other plant hormones in multiple dehydration stresses

Except ABA and JA, 34 orthologs involved in auxins, brassinosteroids, cytokinins, ethylene, gibberellins and salicylic acid biosynthesis and signal transduction exhibited dehydration stress memory (Additional file 7: Table S6). The identified orthologs of auxins biosynthesis and signal transduction were up-regulated, especially in D2. Three orthologs involved in ethylene biosynthesis (Pavir.J11398, Pavir.Ca01179 and Pavir.Ca01180) were up-regulated in D1 and down-regulated in D2. Brassinosteroids, cytokinins, gibberellins and salicylic acid were also participated in the multiple dehydration stresses (Additional file 7: Table S6).

#### Transcription factors (TFs)

In addition to the above TFs (ABFs, JAZs) involved in ABA and JA signal transduction, 46 TFs showed dehydration stress memory in switchgrass, including eight AP2-EREBP genes, six bHLH genes, six MYB/MYB-related genes, six WRKY genes, five NAC genes, four C2H2 genes, three C3H genes and eight other TF genes (Table 2). As with DMGs in general, [+/−] type TFs were the most abundant dehydration memory type, accounting for 47.8% of all dehydration memory TFs. Interestingly, all the dehydration memory WRKY genes belonged to the [+/−] memory type (Table 2).

**Table 2:** Transcription factor involved in transcriptional memory

Gene_id	TF family	C1	D1	D2	DMG types
AP2-EREBP	8				
Pavir.Fb00952	AP2-EREBP	0.42	6.18	16.17	[+/+]
Pavir.Gb00956	AP2-EREBP	6.83	15.62	37.58	[+/+]
Pavir.J10163	AP2-EREBP	5.20	11.52	80.43	[+/+]
Pavir.Ab02556	AP2-EREBP	8.72	18.11	4.74	[+/−]
Pavir.Ia02224	AP2-EREBP	2.74	13.82	1.69	[+/−]
Pavir.Ia04894	AP2-EREBP	4.02	89.68	18.92	[+/−]
Pavir.J08480	AP2-EREBP	8.18	3.87	13.29	[−/+]
Pavir.Aa00834	AP2-EREBP	6.17	1.88	0.39	[−/−]
bHLH	6				
Pavir.J21912	bHLH	5.68	20.24	1.57	[+/−]
Pavir.J10667	bHLH	26.29	12.35	80.85	[−/+]
Pavir.J25904	bHLH	13.71	3.52	8.45	[−/+]
Pavir.Ga02255	bHLH	40.01	16.46	2.57	[−/−]
Pavir.J12143	bHLH	19.24	5.30	1.27	[−/−]
Pavir.J15676	bHLH	8.75	3.21	0.83	[−/−]
MYB/MYB-related	6				
Pavir.Ea02339	MYB	18.01	40.56	0.03	[+/−]
Pavir.Eb02679	MYB	3.60	17.13	0.06	[+/−]
Pavir.J33046	MYB	17.65	35.32	1.17	[+/−]
Pavir.J14839	MYB	28.25	14.11	5.20	[−/−]
Pavir.Ea01343	MYB-related	8.63	3.54	1.25	[−/−]
Pavir.Ea02123	MYB-related	9.62	3.87	1.40	[−/−]
WRKY	6				
Pavir.Bb02088	WRKY	13.42	48.05	23.10	[+/−]
Pavir.Da00323	WRKY	7.21	60.54	0.66	[+/−]
Pavir.Ea02282	WRKY	1.48	6.56	0.18	[+/−]
Pavir.Ea02619	WRKY	4.71	11.20	1.45	[+/−]
Pavir.Ea03280	WRKY	7.13	62.42	1.56	[+/−]
Pavir.J27870	WRKY	25.10	94.49	40.58	[+/−]
NAC	5				
Pavir.J16651	NAC	5.75	21.75	171.04	[+/+]
Pavir.Db00028	NAC	5.04	59.89	0.79	[+/−]
Pavir.J01198	NAC	6.03	34.41	1.49	[+/−]
Pavir.J04751	NAC	0.28	4.48	0.08	[+/−]
Pavir.Ea03233	NAC	29.55	12.72	2.55	[−/−]
C2H2	4				
Pavir.Ia00292	C2H2	0.29	9.29	24.41	[+/+]
Pavir.J00894	C2H2	0.47	26.09	53.69	[+/+]
Pavir.Ia00353	C2H2	5.92	56.11	25.94	[+/−]
Pavir.J25179	C2H2	13.54	91.23	34.93	[+/−]
C3H	3				
Pavir.J04795	C3H	2.76	8.68	27.18	[+/+]
Pavir.J29640	C3H	31.45	186.91	75.98	[+/−]
Pavir.Ea00362	C3H	8.09	2.14	15.60	[−/+]
Others	8				
Pavir.J32718	ARF	7.44	1.93	8.21	[−/+]
Pavir.Ab00132	bZIP	9.16	4.45	0.97	[−/−]
Pavir.Ab03246	C2C2-GATA	9.99	28.48	10.59	[+/−]
Pavir.J02359	G2-like	2.20	4.54	1.01	[+/−]
Pavir.J10950	GRAS	5.86	18.52	5.34	[+/−]
Pavir.Aa02860	HSF	4.49	23.11	61.69	[+/+]
Pavir.J01728	OFP	14.99	6.63	3.07	[−/−]
Pavir.Ba02147	Tify	241.62	115.85	29.98	[−/−]
Numbers of four types of DMGs					
[+/+]	8 (17.4%)				
[−/−]	11 (23.9%)				
[+/−]	22 (47.8%)				
[−/+]	5 (10.9%)				
Total	46				

*C* control, *D1–2* first and second dehydration stresses, R1 first recovery period, “+”, “−” and “=” denote up-regulated genes, down-regulated genes, and genes with no significant changes in expression, respectively

### Osmotic adjustment

**Fig. 7:**
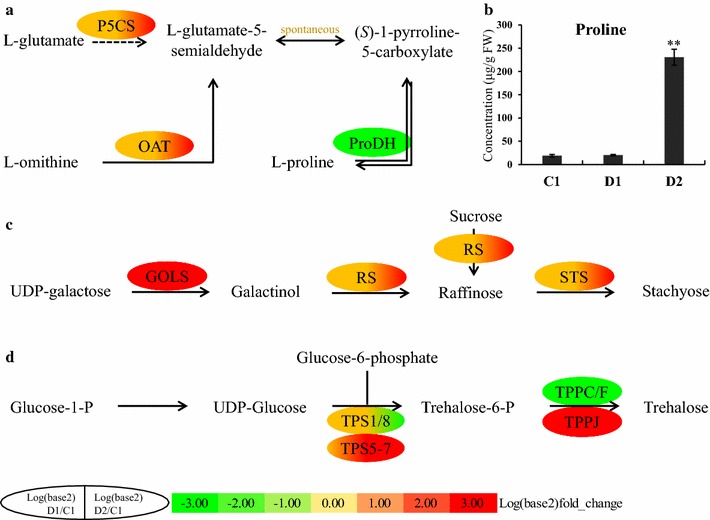
Schematic overview of l-proline, raffinose family oligosaccharide, and trehalose biosynthesis. **a** The biosynthesis of l-proline. **b** The contents of proline in leaves during repeated dehydration stresses. **c** The biosynthesis of raffinose family oligosaccharides. **d** The biosynthesis of trehalose. *P5CS* glutamate 5-kinase [EC 2.7.2.11], *OAT* ornithine aminotransferase [EC 2.6.1.13], *ProDH* proline dehydrogenase [EC 1.5.5.2], *FW* fresh weight, *GOLS* inositol 3-*α*-galactosyltransferase/galactinol synthase [EC 2.4.1.123], *RS* galactinol–sucrose galactosyltransferase/raffinose synthase [EC 2.4.1.82], *STS* galactinol–raffinose galactosyltransferase/stachyose synthetase [EC 2.4.1.67], *TPS* trehalose 6-phosphate synthetase [EC 2.4.1.15], *TPP* trehalose 6-phosphate phosphatase [EC 3.1.3.12]

Osmotic adjustment is essential for water uptake and maintenance, membrane protection, and ROS scavenging in plants subjected to dehydration stress [62]. Switchgrass plants exposed to repeated dehydration stress exhibited differential expression of genes related to proline, raffinose family oligosaccharides (RFO) and trehalose metabolism (Fig. 7; Additional file 8: Table S7). Eight orthologs of P5CS and OAT, the key enzymes involved in the biosynthesis of l-proline from l-glutamate and l-ornithine, respectively, were up-regulated in D2, and an ortholog of proline dehydrogenase (ProDH), which is involved in proline degradation, was down-regulated in D1 and D2 (Fig. 7a; Additional file 8: Table S7). The proline determination assay showed that the proline content of switchgrass leaves had no significant change in D1, but was accumulated in R1 and D2, especially in D2 (Fig. 7b). Several genes related to RFO biosynthesis were up-regulated in D1 and D2, and expressed significantly more strongly in D2, including the *AtGolS3* (AT1G09350) orthologs Pavir.Ba00293 and Pavir.Bb03717, *AtRS2* (AT3G57520) ortholog Pavir.J05624, and *AtRS5* (AT5G40390) ortholog Pavir.Eb00453. All these genes were [+/+] memory type genes. Another four orthologs of *AtRS2* and two orthologs of STS were strongly up-regulated in D2 (Fig. 7c; Additional file 8: Table S7). Among genes related to trehalose biosynthesis, the *AtTPS1* (AT1G78580) ortholog Pavir.Ca01680, Pavir.J00828, and *AtTPS8* (AT1G70290) ortholog Pavir.Ea02872 were down-regulated in D2, while the *AtTPPC* (AT1G22210) ortholog Pavir.J12499, *AtTPPF* (AT4G12430) ortholog Pavir.Ab02625, and Pavir.J38619 were down-regulated in D1 and D2. Additionally, five orthologs of *AtTPS5*-*7* and four orthologs of *AtTPPJ* were up-regulated in D1 and D2, especially during the second stress. Of these genes, the *AtTPS5* (AT4G17770) ortholog Pavir.Ia04010 and *AtTPPJ* (AT5G65140) ortholog Pavir.Fb01190 were [+/+]-type DMGs (Fig. 7d; Additional file 8: Table S7). These results indicate that l-proline, raffinose, stachyose and trehalose play key roles in the resistance of switchgrass to dehydration stress. Moreover, the fact that several [+/+] type memory genes were related to RFO and trehalose biosynthesis demonstrates that switchgrass plants “remember” their first dehydration stress and improve their stress resistance by making a stronger osmotic adjustment during subsequent dehydration stresses.

### Photosynthesis, glycolysis and tricarboxylic acid cycle (TCA cycle)

Physiological assays and pathway enrichment analyses have shown that multiple dehydration stresses cause significant changes in photosynthesis-related energy metabolism and the basic physiological processes that produce ATP and related intermediates that are vital to plant metabolism (Figs. 1f–i; 4; Additional file 4: Table S3). We therefore investigated differentially expressed genes relating to photosynthesis, glycolysis and the TCA cycle. All 28 genes related to the light reactions of photosynthesis (photosystem I, photosystem II, plastoquinol–plastocyanin reductase and ferredoxin-NADP+ reductase) were significantly down-regulated in D2, acting as [=/−] type late-response genes (Additional file 9: Table S8). Among DEGs associated with the C4 photosynthetic carbon assimilation cycle, 15 (78.9%) were down-regulated in D2, and 38 (79.2%) genes related to Calvin cycle were repressed in D2 (Additional file 9: Table S8). These transcriptional data were consistent with the results of physiological PSI and PSII assays using a Dual-PAM 100 instrument (Fig. 1h, i), which indicated that the photosynthetic systems of the switchgrass plants were repressed in D2 and that their behavior during D2 differed significantly from that in D1.

Glycolysis and TCA cycle provide much of the ATP and related intermediates used in plant metabolism. In switchgrass subjected to dehydration stress, nine (90%) orthologs of three key enzymes in glycolysis—hexokinase (EC 2.7.1.1), 6-phosphofructokinase (EC 2.7.1.11) and pyruvate kinase (EC 2.7.1.40)—were up-regulated in D2, indicating that the rate of glycolysis probably increased during the secondary stress even though several other genes related to glycolysis were down-regulated in D2 (Additional file 10: Table S9). In addition, three orthologs of enzymes involved in the first two steps in TCA cycle, citrate (Si)-synthase (EC 2.3.3.1) and aconitate hydratase (EC 4.2.1.3), were up-regulated in D2, while three orthologs of malate dehydrogenase (EC 1.1.1.37) were down-regulated in D2 (Additional file 10: Table S9). These changes in the expression of genes related to glycolysis and the TCA cycle demonstrated that switchgrass plants improved their energy metabolism upon repeated dehydration stress.

### Changes in lignin biosynthesis induced by multiple dehydration stresses

**Fig. 8:**
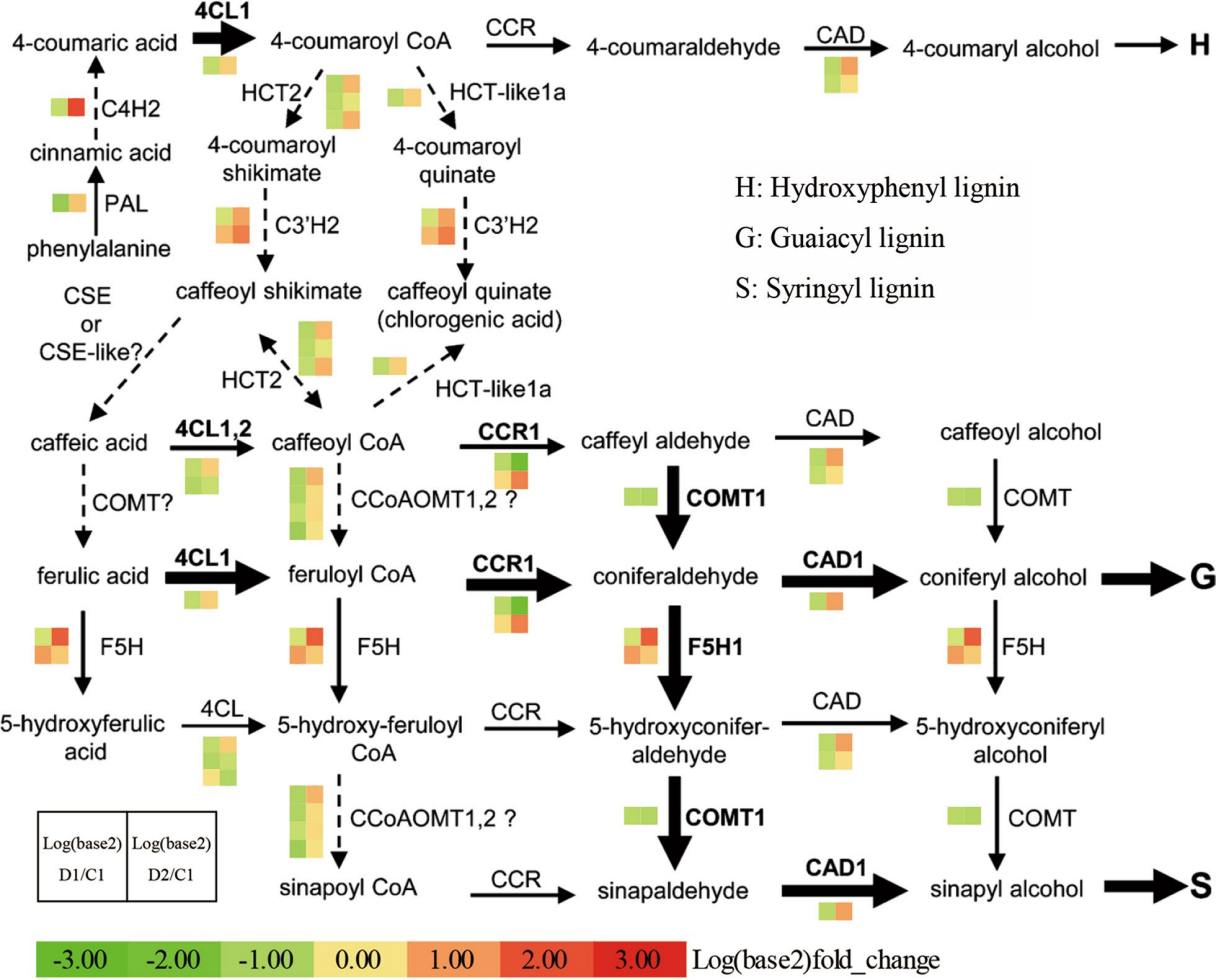
Lignin biosynthesis in multiple dehydration stresses. This model was established based on data from the KEGG database (map00940) and published papers (Shen et al. [63]; Xu et al. [64]; Escamilla-Treviño et al. [65]; Fu et al. [66, 67]). Thicker lines indicate the routes that are most strongly supported by both biochemical and genetic evidence. *PAL* phenylalanine ammonia-lyase, *4CL* 4-coumarate CoA ligase, *C4H* cinnamate 4-hydroxylase, *C3′H* 4-coumaroyl shikimate 3′-hydroxylase, *CCoAOMT* caffeoyl-CoA 3-*O*-methyltransferase, *HCT* hydroxycinnamoyl CoA:shikimate hydroxycinnamoyl transferase, *F5H* ferulate 5-hydroxylase, *CCR* cinnamoyl CoA reductase, *COMT* caffeic acid 3-*O*-methyltransferase, *CAD* cinnamyl alcohol dehydrogenase

Switchgrass is a target lignocellulosic biofuel species, and water-deficit stress strongly affects its biomass accumulation [13, 14]. Pathway enrichment analysis of DMGs in switchgrass plants subjected to repeated dehydration stress revealed enrichment in phenylpropanoid biosynthesis (ko00940) (Additional file 4: Table S3). Based on the published pathway of switchgrass lignin biosynthesis [63–67] and the KEGG database (map00940), we investigated the expression of related genes during the multiple stress treatment. Of the 28 such genes that were detected, ten were differentially expressed: *PvPAL1* and *PvCCoAOMT2b* were down-regulated in D1 and up-regulated in D2, belonging to the [−/+] memory gene type; eight genes were identified as [=/+] late-response genes, including *PvC4H2*, *PvC3′H1a*, *PvCCoAOMT1a*, *PvHCT1a*, *PvHCT2a*, *PvF5H1*, *PvCCR*-*like1a* and *PvCAD1* (Fig. 8; Additional file 11: Table S10). These results demonstrate that lignin biosynthesis in switchgrass was repressed in D1 but increased in D2.

### Persistence of transcriptional memory

**Fig. 9:**
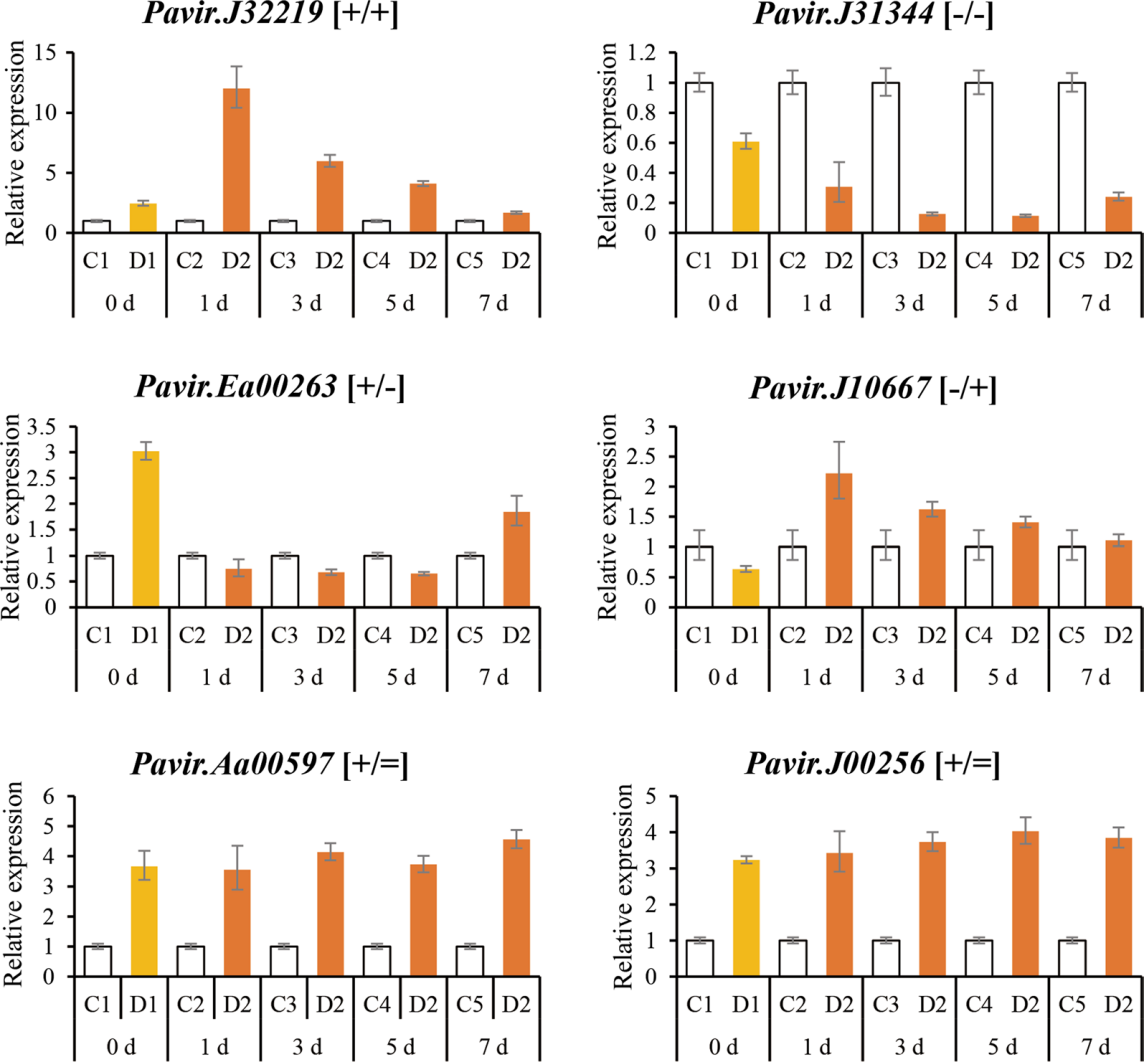
Length of the transcriptional memory detected by quantitative real-time PCR. *C* control, *D1–2* the first and second dehydration stresses. Relative gene expressed levels were calculated using the ΔΔ*C*_T_ method with *PveEF*-*1α* as the internal control, and three biological replicates were performed for each experiment

To investigate the persistence of the transcriptional memory in the absence of dehydration stress, trained and untrained plants were replanted in soil for 3, 5, and 7 days (Fig. 1b), and the expression of four representative memory genes and two non-memory genes was monitored by qRT-PCR. After 3 and 5 days, the expressions of the memory genes in trained plants subjected to a second dehydration stress event (D2) differed significantly from those of non-trained plants subjected to their first dehydration stress (D1). However, the expression of these genes in trained plants started to return towards the baseline level after 7 days. The relative expression of the non-memory genes in D2 was always similar to that in D1 (Fig. 9). These results showed that dehydration stress memory persists for around 5 days in switchgrass plants, but gradually fades after 7 days in water.

### Dehydration memory genes of switchgrass and maize

Dehydration memory has been observed in maize and *Arabidopsis*, and is considered to be a common phenomenon in plants [30, 31]. To better characterize the conservation and species-specificity of dehydration memory genes, we investigated the similarities and differences between dehydration memory in switchgrass, maize and *Arabidopsis*. In total, 47,207 genes were detected in the switchgrass transcriptomes, which was more than in maize (39,635) or *Arabidopsis* (33,555). There were 1566 (3.3%) and 5639 (11.9%) switchgrass genes that were differentially expressed in D1 and D2; the corresponding values for maize were 2062 (5.2%) and 2924 (7.4%), while those for *Arabidopsis* were 6579 (19.6%) and 1371 (4.1%) (Table 1). The number of response genes in switchgrass was similar to that in maize, but significantly less than in *Arabidopsis*. In switchgrass, 47.3% (741) of all dehydration response genes were identified as DMGs, while the ratio was 39.6% (816) in maize and 29.8% (1963) in *Arabidopsis*. Remarkably, the 51% of DMGs were [+/−] type memory genes, which is similar to the proportions in maize (65%) and *Arabidopsis* (44%) (Table 1).

**Fig. 10:**
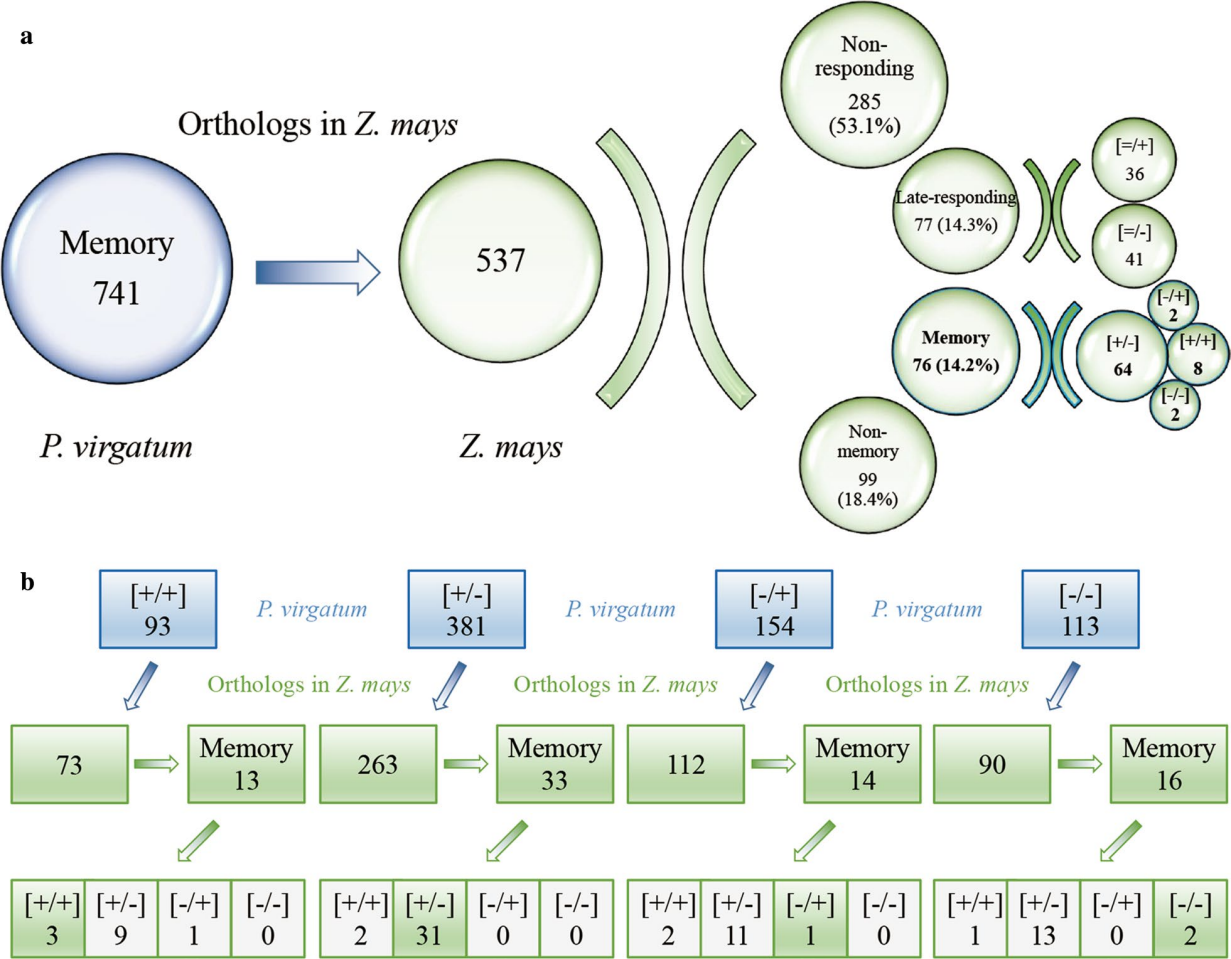
Orthologous compare of response patterns to multiple dehydration stress in switchgrass and maize. **a** Comparison of orthologous dehydration memory genes in switchgrass and maize. **b** Distribution of switchgrass orthologs in maize according to their memory response types

To clarify the functions of DMGs and identify key genes involved in dehydration memory, we compared switchgrass DMGs to maize and *Arabidopsis* databases using the Blast tools. Few DMGs in switchgrass were homologous to proteins in the TAIR10 *Arabidopsis* database, so the following section focuses on similarities and differences between switchgrass and maize. 741 memory genes had 537 orthologs in maize, including 76 (14.2%) DMGs, 99 (18.4%) non-memory genes, 77 (14.3%) late-responding genes and 285 non-responding genes. Among these 76 DMGs, 64 (84.2%) were [+/−] type genes (Fig. 10a).

We then investigated whether the orthologs of memory genes in switchgrass displayed similar types of memory responses in maize. The maize orthologs of the switchgrass memory genes included both conserved and non-conserved memory types, but the orthologs belonged to other types (non-memory genes, late-responding genes and non-responding genes) were the largest categories (Fig. 10b). Every type of switchgrass memory genes had orthologs belonging to the same memory type in maize, even though the numbers were significantly different: 3 [+/+], 31 [+/−], 1 [−/+] and 2 [−/−]. Remarkably, almost all the [+/−] type orthologs (93.9%) belonged to the same memory type (Fig. 10b; Additional file 12: Table S11). These conserved memory genes probably played important roles in the multiple stress response and are involved in plant growth, signal transduction, and other unknown pathways (30.2%). In addition, many memory orthologs belonged to different memory types (48.7%) in the two species, demonstrating the evolution of species-specific transcriptional memory responses (Additional file 13: Table S12).

## Discussion

### Biological significance of transcription memory in switchgrass

In natural environments, plants may suffer water-deficit stress on several occasions over a few days [68]. In this study, we designed experiments to alter the water balance with successive cycles of water-deficit stresses. We hypothesized that dehydration stress memory in switchgrass helped the trained plants to resist subsequent water-deficit stresses with stronger and faster dehydration responses.

Several gene families were found to include coexisting dehydration stress memory genes, non-memory genes, and late-response genes. This has also been observed in both *Arabidopsis* and maize [30, 31]. For instance, nine orthologs of NCED were detected by RNA-Seq, four of which were differentially expressed in D1 and D2. However, only one (Pavir.Ba03791) was a [+/+] memory gene, i.e., a gene expressed more strongly in D2 than in D1 (Additional file 5: Table S4). Trehalose 6-phosphate synthetase (TPS) is the key enzyme in trehalose biosynthesis [69]. Of its eight differentially expressed orthologs in switchgrass, three were down-regulated ([=/−]) between D1 and D2, four were up-regulated (=/+) between D1 and D2, and one was a [+/+] memory gene (Additional file 8: Table S7). These results suggest that the orthologs have developed different functions during evolutionary history, with several members of the family providing stress memory to reduce energy consumption and improve resistance to multiple drought stresses.

### Functions of ABA in dehydration stress memory

ABA is strongly induced by drought stress and plays key roles in the drought stress response [17, 70]. In *Arabidopsis*, endogenous ABA accumulated in D1, became less abundant in R1, and accumulated again in D2. However, the extent of its accumulation in D1 was identical to that in D2 [28]. After 7 days of watering, ABA levels could be restored to their prior peak despite the loss of dehydration stress memory [28]. In the *areb1/areb2/abf3 Arabidopsis* triple mutant, the expressed levels of trained marker genes were strongly reduced but remained much higher than the transcript level in D1 [38]. These results indicate that ABA is not the key factor in the induction of transcriptional memory in *Arabidopsis*. In this work, ABA accumulated in D1, R1 and D2, and especially strongly in R1 and D2 (Fig. 5d). In contrast to the observations for *Arabidopsis*, there was significant ABA accumulation during R1 and D2 in plants subjected to repeated dehydration stress, indicating that ABA may play different roles in switchgrass and *Arabidopsis,* and revealing a species-specific differences between switchgrass (a C4 monocot plant) and *Arabidopsis* (a C3 eudicot plant).

### JA accumulation and its crosstalk with ABA

JA is an important hormone that plays key roles in plant responses to biotic and abiotic stresses [61, 71]. It is synthesized rapidly when a plant suffers drought, cold or other stresses [59, 72]. In switchgrass, JA-related genes (especially downstream genes expressed in the peroxisome) were significantly up-regulated in D2 (Fig. 5b; Additional file 6: Table S5). This suggests that JA may play an important role in the response to repeated dehydration stresses. However, HPLC–MS data suggested that JA was accumulated in D1 but recovered to its initial levels in R1 and D2 (Fig. 5d). It is not clear why JA accumulated in D1 but not in D2, because the downstream genes of JA biosynthesis were up-regulated in D2.

JA is synthesized from α-linolenic acid in chloroplasts, and the step that commits the substrate to JA formation is the formation of 13-hydroperoxy-9,11,15-octadecatrienoic acid (13-HPOT) catalyzed by allene oxide synthase (AOS) [61]. The expression of AOS is controlled by a bHLH transcriptional factor, MYC2 [40]. In switchgrass, two orthologs of MYC2 were slightly up-regulated in D1 and down-regulated in D2, and another four orthologs of MYC2 were down-regulated in both D1 and D2 (Fig. 6; Additional file 6: Table S5). Previous studies revealed that transient JA accumulation is needed for the increase in ABA levels in citrus roots under drought stress [60]. Therefore, we inferred that when the switchgrass plants experienced the first dehydration stress, *MYC2* was rapidly up-regulated and induced the expression of *AOS*, leading to the quick accumulation of JA. Conversely, during secondary dehydration stress, all the orthologs of MYC2 were down-regulated, suppressing the transcription of *AOS* and thus reducing JA accumulation even though the downstream genes of JA biosynthesis were up-regulated.

There is sophisticated crosstalk between JA and ABA, with both synergistic and antagonistic effects in plants. For example, dehydration stress and ABA can induce JA synthesis [73–75], and ABA plays a key role in inducing MYC2 expression in response to JA in *Arabidopsis* [40]. Conversely, in citrus roots, increased ABA levels were observed following transient JA accumulation [60]. In this work, JA accumulated during D1 and ABA accumulated in D1, R1 and D2, especially in the latter two periods (Fig. 5d). These results suggest that the transient accumulation of JA may induce strong ABA accumulation, but ABA accumulation is not sufficient to induce JA accumulation. In addition, MYC2 is critical for JA biosynthesis but it was first identified as a component of an ABA signal transduction system [17, 40], suggesting that it may play an important roles in the crosstalk between JA and ABA during repeated dehydration stress (Fig. 6). Further studies on this signaling network are warranted.

### Osmotic adjustment, energy metabolism and lignin biosynthesis

Osmotic adjustment is a common plant response to water-deficit stress, but plant species differ in their patterns of osmolyte biosynthesis. For instance, proline, raffinose and galactinol accumulated in *Arabidopsis* and provided protection against drought, salinity, and cold stress [76, 77]. Conversely, raffinose and trehalose (but not proline) were synthesized in the leaves of the physic nut under severe drought stress [45]. In switchgrass subjected to dehydration stress, genes related to the biosynthesis of proline, raffinose family oligosaccharides, and trehalose were strongly up-regulated in leaves (Fig. 7; Additional file 8: Table S7), indicating that these substances play important roles in osmotic adjustment, especially in secondary dehydration stress. The results of proline content determination assay demonstrated that proline was significantly accumulated in D2, and these data are consistent with the related gene expression (Fig. 7a, b).

Plants may adjust their energy metabolism to increase the supply of ATP and related compounds [45, 78]. The physiological assays conducted in this work and the transcriptional data for PSI and PSII showed that photosynthesis and the Calvin cycle proceeded normally in D1, but were significantly suppressed in D2 (Fig. 1; Additional file 9: Table S8). In addition, orthologs of several key enzymes in glycolysis and the TCA cycle were differentially expressed during secondary dehydration stress, causing changes in the ATP supply and intermediate metabolism. The affected enzymes include hexokinase, 6-phosphofructokinase, pyruvate kinase, citrate (Si)-synthase, aconitate hydratase and malate dehydrogenase (Additional file 10: Table S9). The different responses during D1 and D2 demonstrate the transcriptional memory of switchgrass.

Switchgrass is a lignocellulose biofuel plant, and genes important in its biosynthesis of lignin have been identified in vivo and in vitro [63]. During drought stress, microRNAs regulate its lignin and biomass synthesis [14, 79], and the total plant lignin concentrations increased during drought [80]. In this work, genes related to lignin biosynthesis were slightly down-regulated in D1 but significantly up-regulated in D2, and thus exhibited [=/+] or [−/+] memory behavior (Fig. 8; Additional file 11: Table S10). The data on the transcription of lignin biosynthesis genes acquired in this work will provide an excellent foundation for the genetic modification of switchgrass.

### Dehydration stress memory in *Arabidopsis*, maize and switchgrass

Transcriptional memory persisted for ~ 5 days in *Arabidopsis* [28] and maize [31], and was associated with trimethylated histone H3 Lys4 (H3K4me3) and a stalled RNA polymerase II [28]. In this work, the expressions of representative dehydration stress memory genes recovered to their initial levels after 7 days of rewatering (Fig. 9). It this appears that transcriptional memory in switchgrass persists for ~ 5 days, which is enough to accommodate the diurnal cycle of dehydration stress, and probably plays important roles in the response to long-term successive dehydration stresses.

Dehydration stress memory has been observed in several species, but DMGs were identified only in *Arabidopsis* and maize [30, 31]. In this study, we identified DMGs in switchgrass, and identified interesting similarities and differences between these three species: (i) the number of response genes in switchgrass was similar to that in maize, but much lower than that for *Arabidopsis*, whereas the number of late-response genes in switchgrass was significantly greater than in the other two species. (ii) The ratio of DMGs to all response genes in switchgrass (47.3%) was higher than the ratios in maize (39.6%) and *Arabidopsis* (29.8%). (iii) Orthologs of switchgrass DMGs in maize mainly belonged to other gene types (non-response, non-memory, or late-response genes); even those that contributed to dehydration stress memory in two species generally functioned as different types of memory gene in each. (iv) 14.2% of all orthologs of switchgrass DMGs in maize exhibited dehydration stress memory, and each type had orthologs belonging to the same memory type in maize. Nearly all the orthologs (93.9%) of [+/−]-type genes belonged to the same memory type in maize. These memory-conserved genes demonstrated the conservation of dehydration stress memory during evolution, and are likely to be excellent targets for genetic modification in switchgrass or related crops in the family Gramineae (Additional file 12: Table S11). With the methods of overexpression, drought-induced expression or gene editing, the [+/+] and [−/−] memory genes are considered as excellent targets to enhance drought tolerance of switchgrass and other crops.

## Conclusions

In this study, we identified 741 dehydration stress memory genes in switchgrass plants subjected to multiple dehydration stresses. Analyses of specific pathways combined with physiological data allowed us to explore the molecular mechanisms of repeated dehydration stress responses in switchgrass at the transcriptional and physiological levels. Switchgrass plants acclimatized to secondary dehydration stresses by regulating the crosstalk of ABA and JA biosynthesis and signal transduction, adjusting the biosynthesis of osmotic substances (l-proline, stachyose and trehalose), changing their energy metabolism (by modifying the expression of genes relating to photosynthesis systems, glycolysis, and the TCA cycle), and through lignin biosynthesis. The transcriptional data showed that dehydration memory persisted for ~ 5 days in switchgrass. Analyses of homologous genes in switchgrass, maize and *Arabidopsis* revealed that some memory genes are conserved across species, but there are also important species-specific aspects of stress memory. These results revealed a previously unknown transcriptional memory behavior in switchgrass, and the genes and pathways identified in this study will be useful for the future studies of switchgrass and other crops.

## Additional files

**Additional file 1: Figure S1.** Overview of the RNA-Seq results and repeatability of different biological replicates. The first eight figures show the proportion of clean reads in the sequenced samples; the next three figures show the repeated expression of genes in two biological replicates; the last four figures showed the correlations between differentially expressed genes in two biological replicates. C, control; D1-2, the first and second dehydration stresses; R1, the first recovery period.

**Additional file 2: Table S1.** List of dehydration memory genes (DMGs) identified in this work.

**Additional file 3: Table: S2.** Primers used in qRT-PCR.

**Additional file 4: Table S3.** Top 20 pathways involved in dehydration memory.

**Additional file 5: Table S4.** Differentially expressed genes involved in ABA biosynthesis, degradation and signal transduction.

**Additional file 6: Table S5.** Differentially expressed genes involved in JA biosynthesis and signal transduction.

**Additional file 7: Table S6.** Differentially expressed genes involved in other plant hormones biosynthesis and signal transduction.

**Additional file 8: Table S7.** Differentially expressed genes involved in the biosynthesis of L-proline, stachyose and trehalose.

**Additional file 9: Table S8.** Differentially expressed genes involved in photosynthesis.

**Additional file 10: Table S9.** Differentially expressed genes involved in glycolysis and TCA cycle.

**Additional file 11: Table S10.** Differentially expressed genes involved in lignin biosynthesis.

**Additional file 12: Table S11.** Common DMGs in switchgrass and maize.

**Additional file 13: Table S12.** List of DMGs that are different between switchgrass and maize.

## Abbreviations

ABA: abscisic acid
BLAST: basic local alignment search tool
cDNA: complementary DNA
D1–3: the first to third dehydration stress
DEG: differentially expressed genes
DMG: dehydration stress memory gene
FPKM: fragments per kilobase of exon per million fragments mapped
GO: Gene Ontology
HPLC–MS: high performance liquid chromatography–mass spectrometry
JA: jasmonic acid
KEGG: Kyoto encyclopedia of genes and genomes database
nr: NCBI non-redundant protein sequences database
PSI and PSII: photosynthesis system I and II
qN: non-photochemical quenching
qRT-PCR: quantitative real-time PCR
R1–2: the first and second dehydration stress
RGAP: rice genome annotation project
RNA-Seq: RNA sequencing
RWC: Relative water content
TAIR10: *Arabidopsis* information resource proteins database
TCA cycle: tricarboxylic acid cycle
