# Supplementary figures and images for: Transcriptional and physiological data reveal the dehydration memory behavior in switchgrass (Panicum virgatum L.)
Source: Biotechnol Biofuels. 2018 Apr 2;11:91. doi: 10.1186/s13068-018-1088-x (PMC5879616; doi:10.1186/s13068-018-1088-x)

**Figure S1 The overview of RNA-Seq and repeatability of different biological replicates**


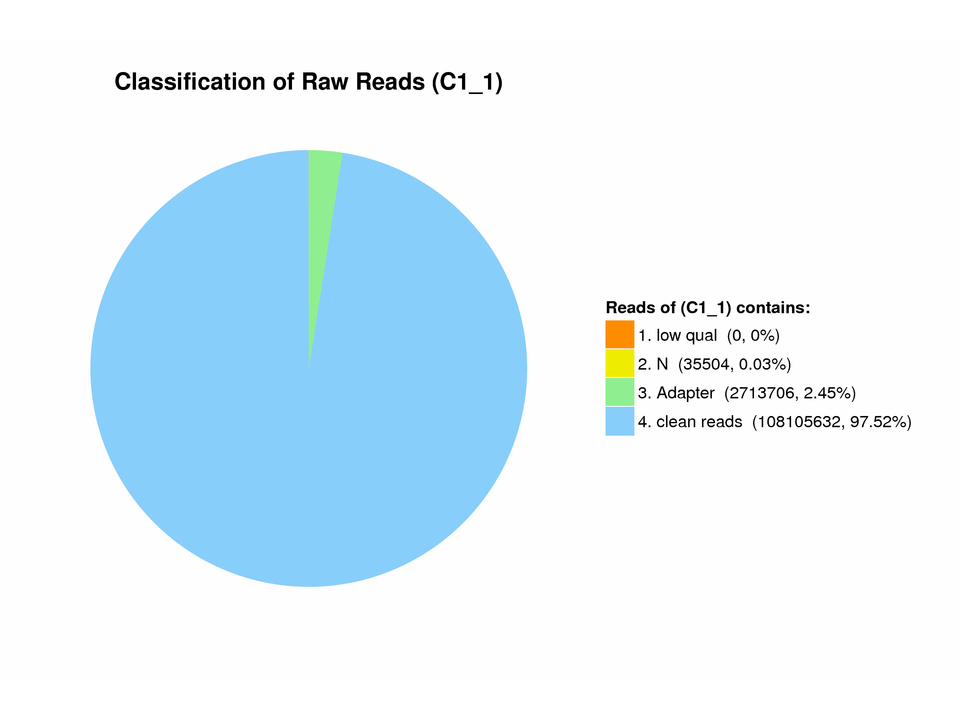

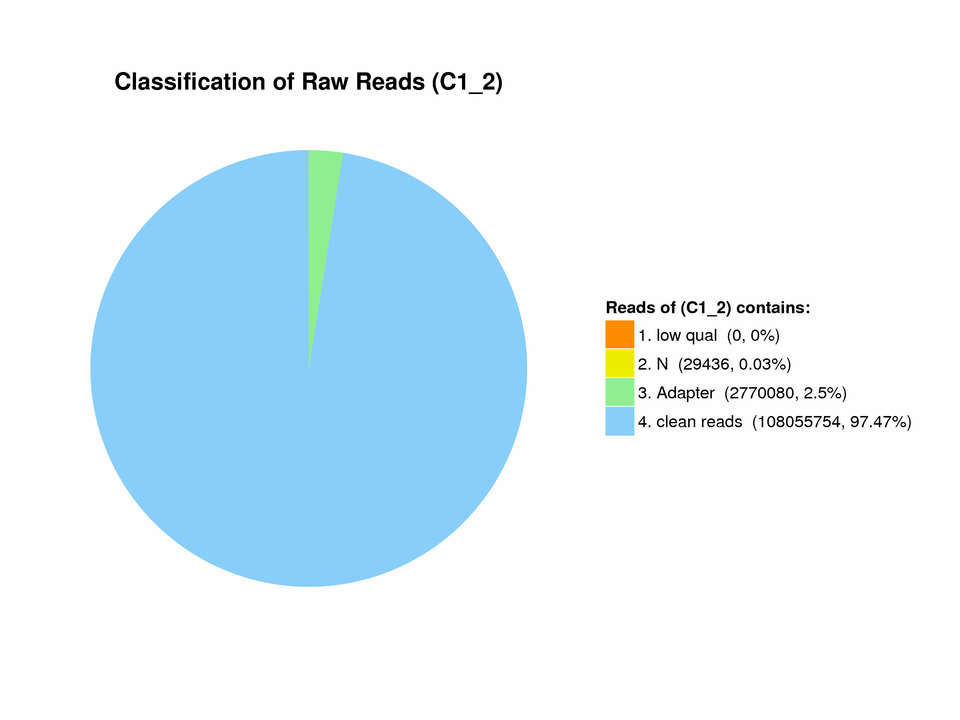

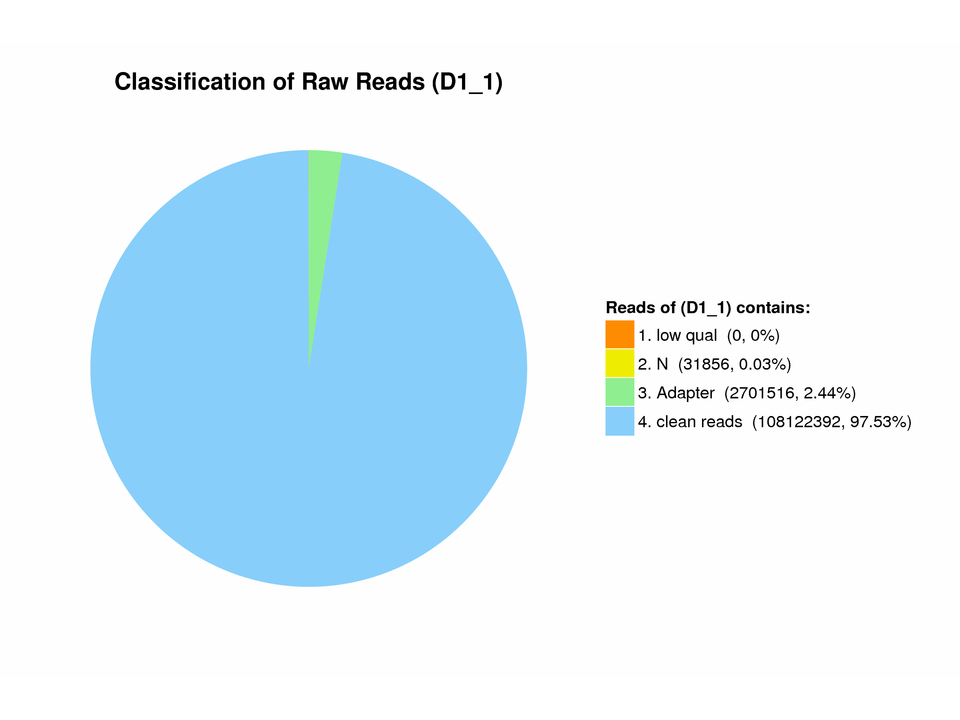

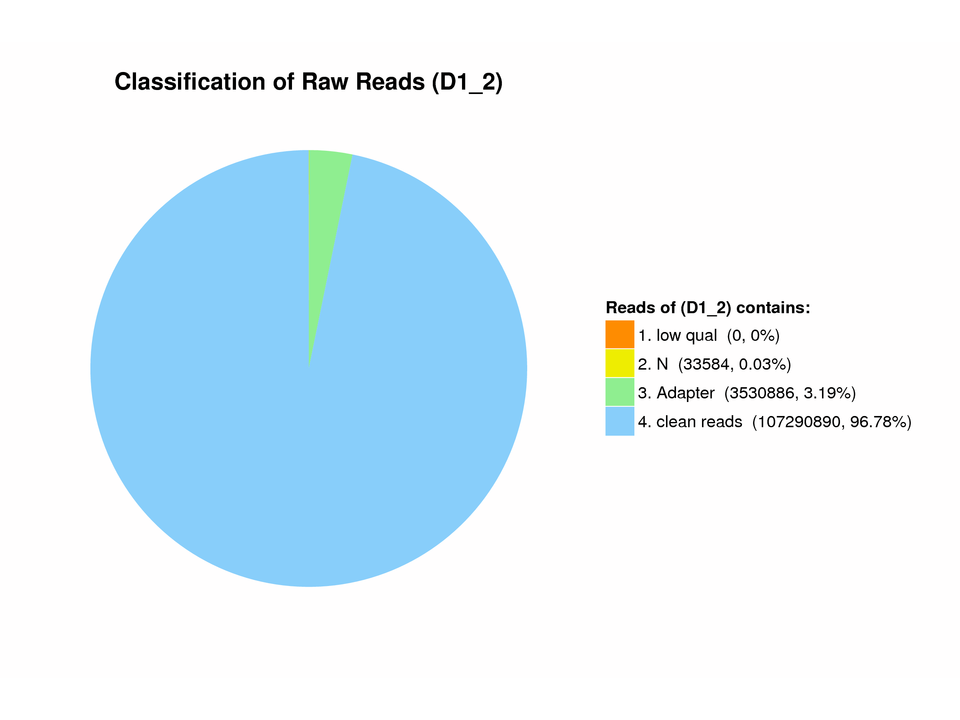

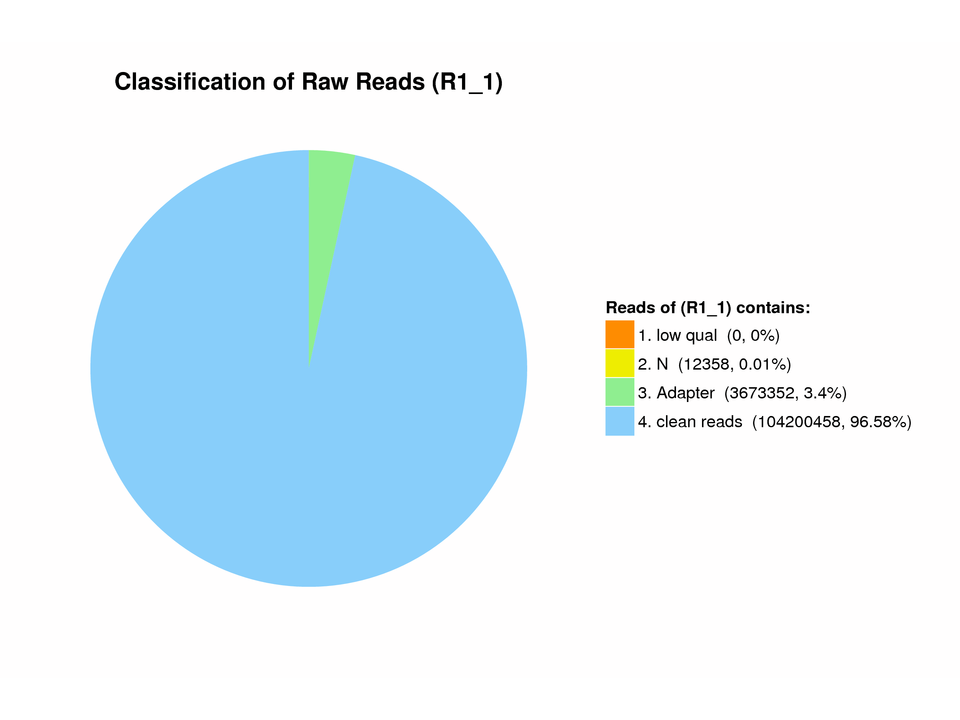

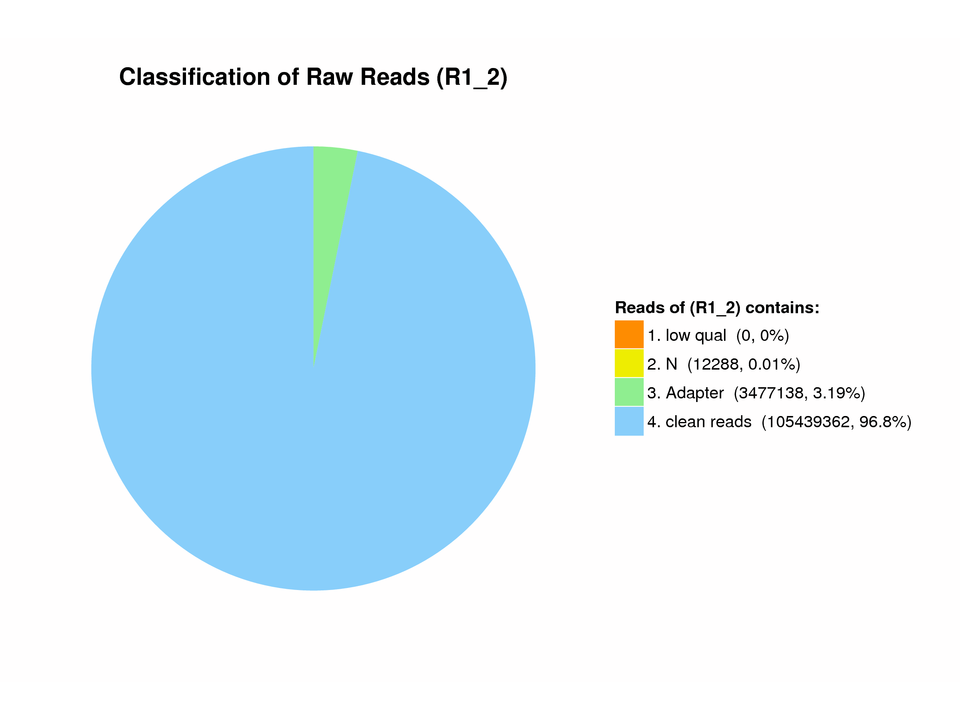

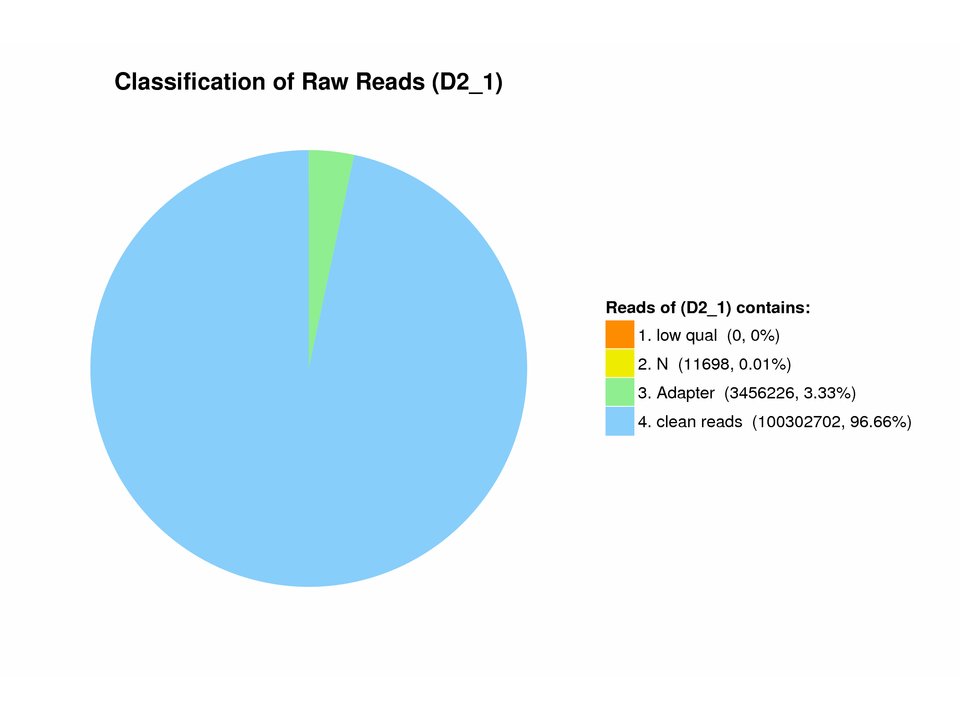

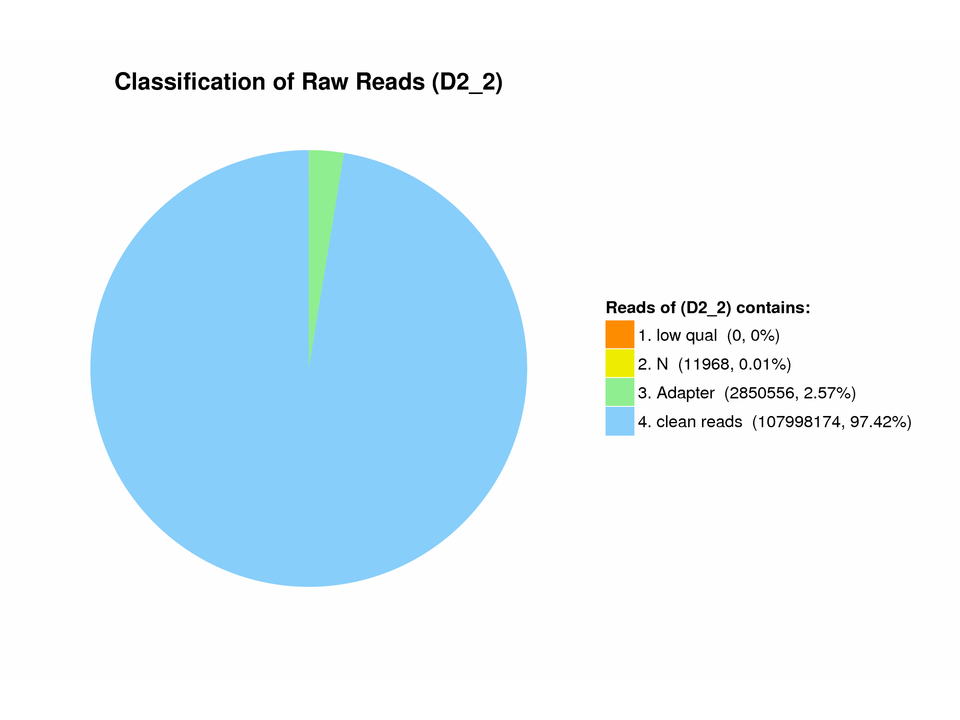

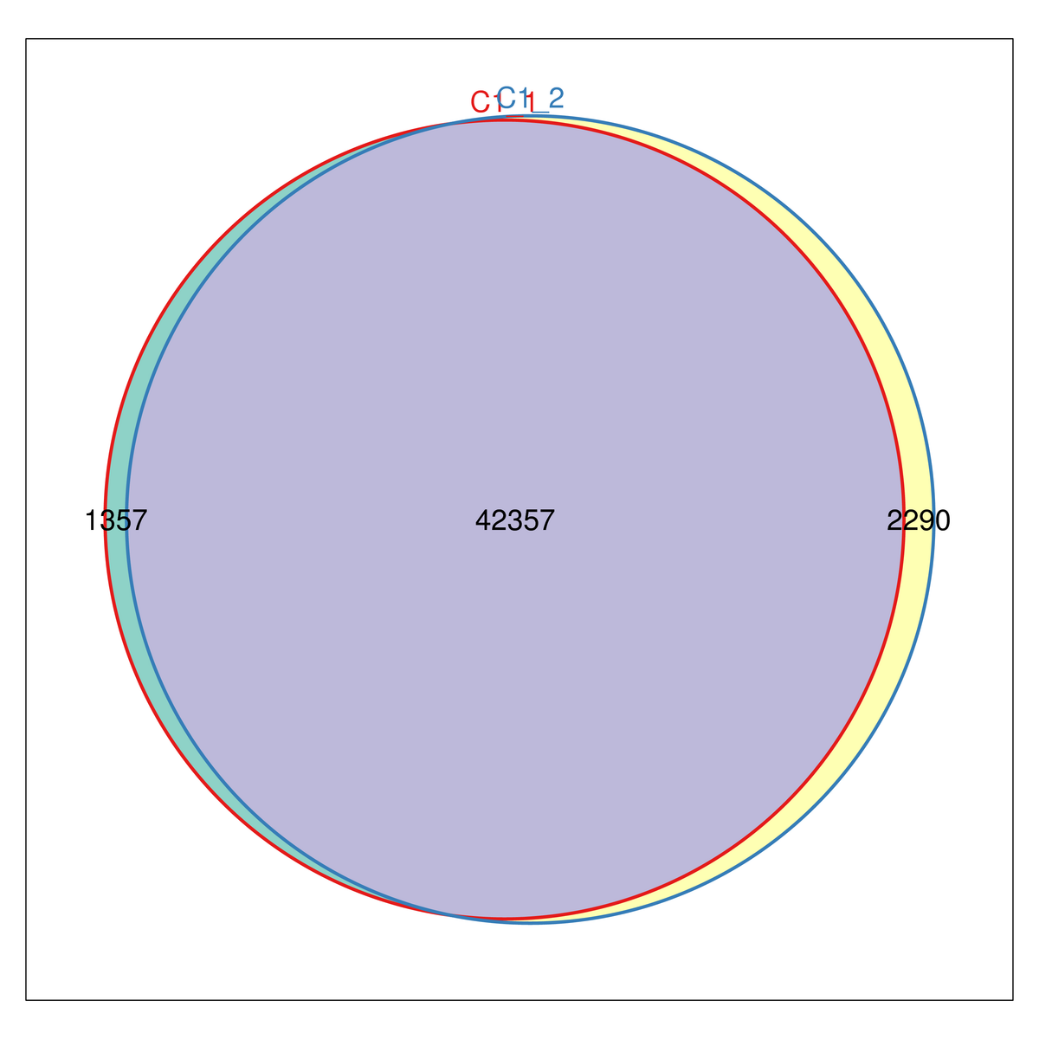

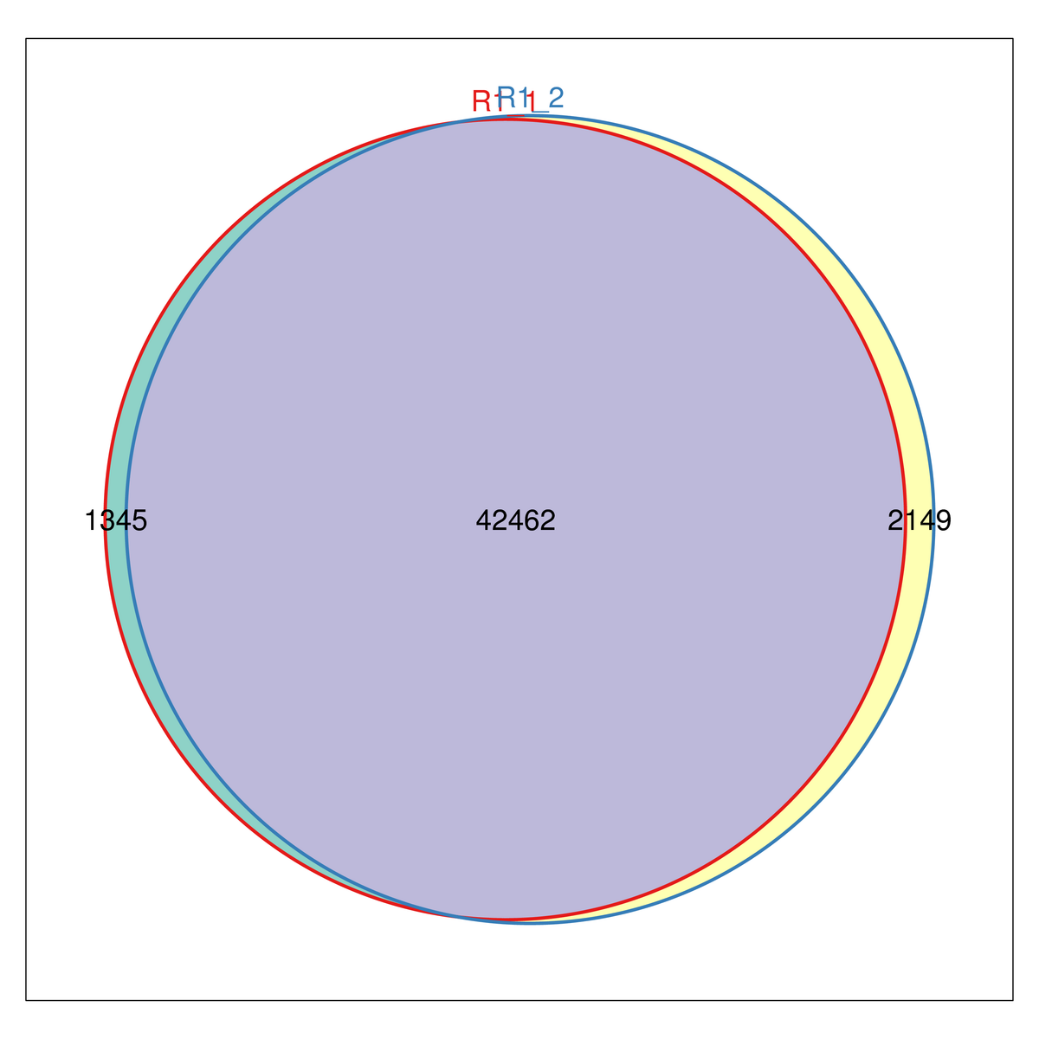

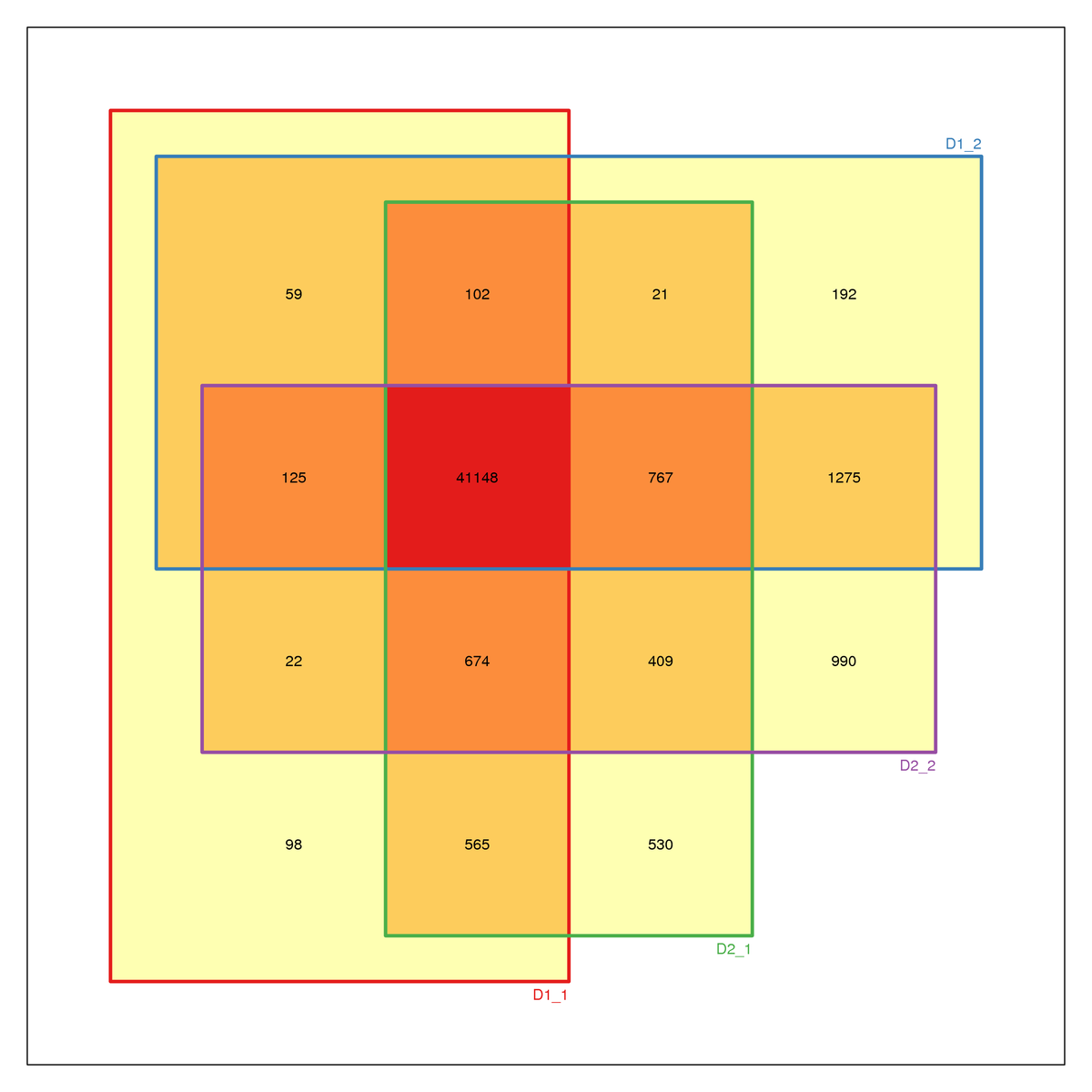


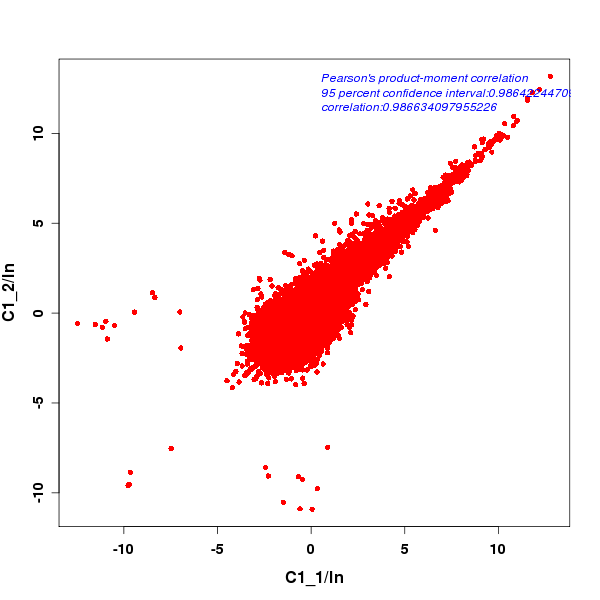

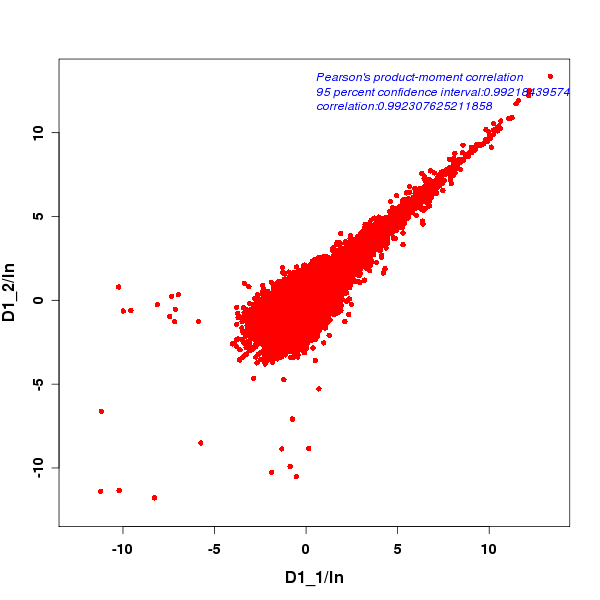

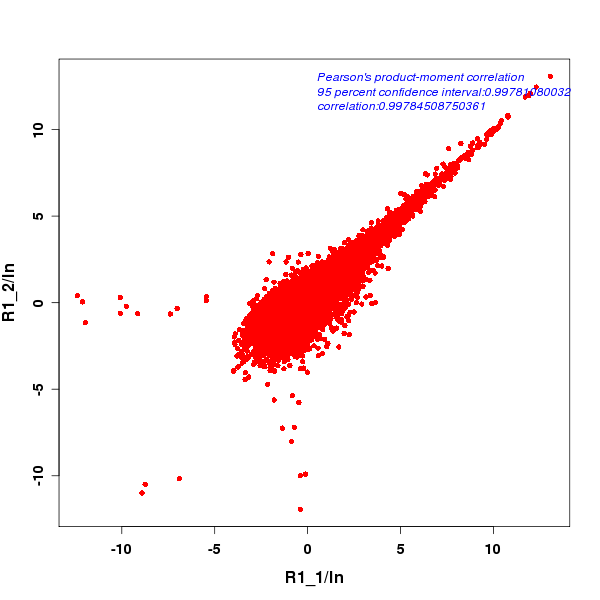

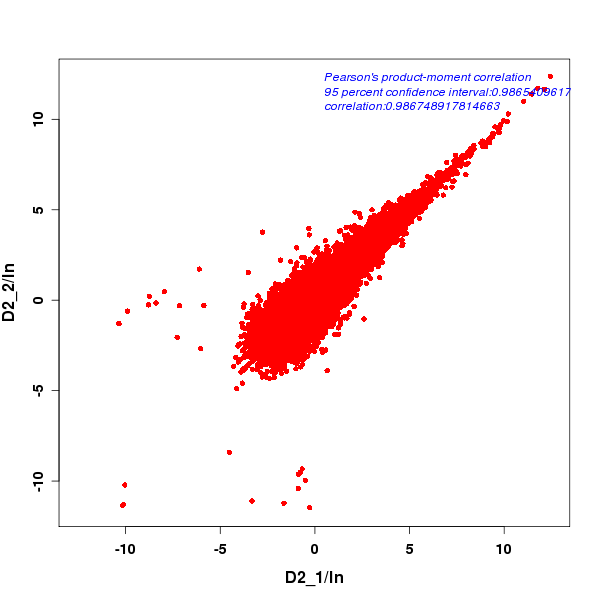

Supplement: Supplementary file 1 — Additional file 1: Figure S1. Overview of the RNA-Seq results and repeatability of different biological replicates. The first eight figures show the proportion of clean reads in the sequenced samples; the next three figures show the repeated expression of genes in two biological replicates; the last four figures showed the correlations between differentially expressed genes in two biological replicates. C, control; D1-2, the first and second dehydration stresses; R1, the first recovery period. [file 13068_2018_1088_MOESM1_ESM.docx]
